# Does a Biochemical Approach Facilitate the Diagnosis of Prenatal Alcohol Exposure and Fetal Alcohol Spectrum Disorder in Neonatal Period?

**DOI:** 10.3390/ijms27104357

**Published:** 2026-05-14

**Authors:** Iwona Jańczewska, Marek Wiergowski, Jolanta Wierzba, Monika Cichoń-Kotek, Mateusz Kacper Woźniak, Marek Biziuk

**Affiliations:** 1 Department of Neonatology, Faculty of Medicine, Medical University of Gdansk, Mariana Smoluchowskiego 17 Street, 80-214 Gdansk, Poland; 2 Department of Forensic Medicine, Faculty of Medicine, Medical University of Gdansk, Debowa 23 Street, 80-211 Gdansk, Poland; marwier@gumed.edu.pl (M.W.);; 3 Division of Internal and Pediatric Nursing, Faculty of Health Sciences with the Institute of Maritime and Tropical Medicine, Medical University of Gdansk, Debinki 7 Street, 80-211 Gdansk, Poland; 4 Department of Pediatrics, Haematology and Oncology, Faculty of Medicine, Medical University of Gdansk, Debinki 7 Street, 80-952 Gdansk, Poland; 5 Department of Analytical Chemistry, Faculty of Chemistry, Gdansk University of Technology, Gabriela Narutowicza 11/12 Street, 80-233 Gdansk, Poland

**Keywords:** fetal alcohol spectrum disorder (FASD), fetal development, fetal growth restriction (FGR), postnatal growth impairments, neurodevelopmental disorders, epigenetic modifications, environmental exposure, genetic factors

## Abstract

Prenatal alcohol exposure (PAE) can cause fetal alcohol spectrum disorder (FASD). The FASD continuum encompasses facial dysmorphism, growth failure, and central nervous system (CNS) abnormalities/dysfunctions. Because some of these features may not be apparent in newborns, detecting PAE in the neonatal period is challenging, while early diagnosis may improve neurodevelopmental outcomes. Maternal self-reported alcohol consumption is limited by recall bias and denial, leading to misdiagnosis. Currently, there is a lack of universally implemented and standardized tools for identifying PAE/FASD in children across clinical settings. We aimed to review the existing literature on PAE assessment methods. Analysis of alcohol metabolites in neonatal meconium is the most widely studied and appears to be feasible for routine use, but it has some limitations. Recent advances in understanding the effects of alcohol on neurotransmitters, growth factors, and gene activity have contributed to the development of novel diagnostic strategies and have brought us closer to effective PAE detection. Some laboratory assays appear to be feasible for implementation in routine clinical practice, i.e., testing for pro- and anti-inflammatory cytokines, including interleukins (IL): IL-6, IL-1β, IL-10, and tumor necrosis factor-alpha (TNF-α) and Insulin-like Growth Factor 1(IGF1). These molecular approaches hold promise but require replication and validation before becoming the standard in clinical practice. Further research on biomarkers and other screening tools should continue to determine their feasibility and availability.

## 1. Introduction

Prenatal alcohol exposure (PAE) may result in damage to the developing brain and other fetal organs, leading to the occurrence of one of many forms of fetal alcohol spectrum disorder (FASD) in the exposed child. The FASD continuum encompasses a range of morphological and neurodevelopmental abnormalities. Within this spectrum, full-blown fetal alcohol syndrome (FAS), partial fetal alcohol syndrome (pFAS), alcohol-related neurodevelopmental disorder (ARND), and alcohol-related birth defects (ARBD) are recognized [1,2,3]. FASD is a devastating condition that profoundly impacts the lives of individuals and their families. Therefore, early recognition of PAE and FASD is crucial for providing appropriate support to children and their families, improving their neurodevelopmental outcomes, and daily and social functioning.

Since the first descriptions of FASD, many researchers have sought effective methods for diagnosing FASD and related disorders. In 1996, a committee of the Institute of Medicine (IOM) delineated diagnostic standards for FAS, based on clinical criteria. Moreover, the committee set the direction for public health care to meet the needs associated with this condition [1]. Various clarifications and modifications of the IOM criteria have been developed to assist healthcare professionals in making a clinical diagnosis [4,5,6]. Efforts have been made to develop guidelines that best meet the needs of individual countries [7,8,9,10]. Most guidelines are based on a set of symptoms of varying severity, including the presence of characteristic facial dysmorphic features, growth failure (either prenatally, later in life or both), and at least one structural central nervous system (CNS) abnormality, e.g., microcephaly and/or neurobehavioral dysfunction [11,12]. According to the IOM’s 2016 updated criteria, the syndrome can be diagnosed with or without PAE [5]. The most characteristic facial features include short palpebral fissures, a smooth philtrum, and a thin upper vermillion border [3,11,12]. Diagnosing this syndrome in the neonatal period and early childhood can be challenging because even heavily exposed newborns may not exhibit facial dysmorphism or growth deficiency [13]. Furthermore, functional abnormalities of the CNS may be absent or subtle in the first year of life, while adolescents almost always exhibit disorders of behavior, attention, and executive functions (higher cognitive adaptive processes) [3,12]. They are often referred to neurologists, psychiatrists or psychologists with suspicion of other disorders, such as attention deficit/hyperactivity disorder (ADHD) or intellectual, verbal or cognitive impairments. It has been shown that up to 80% of these patients had PAE as the underlying cause of these problems [14].

Healthcare professionals are increasingly aware of the typical signs of FAS. However, recognizing cases without characteristic facial dysmorphic features requires knowledge and experience, a thorough understanding of diagnostic scales, and sometimes the use of advanced approaches, such as photography combined with computer analysis [15]. Such tools are usually not available outside research settings. Diagnosing neurobehavioral, cognitive, and speech disorders requires the involvement of a multidisciplinary panel consisting of pediatricians, neuropsychologists, speech-language pathologists, physical and occupational therapists, social workers and other trained professionals. However, this approach is expensive, time-consuming, and inaccessible to many children, leading to underdiagnosis and misdiagnosis [3,16]. Therefore, FASD is diagnosed at various stages of the patient’s life, and only a fraction of those affected are diagnosed before they reach school age [5,7,11].

The first step in the FASD diagnostic process should be identifying a group of children prenatally exposed to alcohol so that they can be referred for early specialized evaluation for FASD. This is a key element for early diagnosis and, therefore, for the implementation of therapeutic interventions.

The standard for detecting fetal alcohol exposure remains a maternal self-report of alcohol consumption. Currently, the PAE assessment in many obstetric and neonatal care facilities relies on routine questions about alcohol consumption, asked by a physician or midwife during prenatal visits or upon admission to the hospital for delivery [17]. Alternatively, tools specifically developed and validated for screening for alcohol use in pregnant women, such as the Alcohol Use Disorders Identification Test—consumption subset (AUDIT-C) or the Timeline Follow-Back (TLFB) interview, are used [18,19]. The AUDIT-C consists of three short questions about the frequency, number of standard drinks, and consumption of 5 or more drinks on a single occasion. Each question has five responses and is scored from 0 to 4. Points are summed for each question, resulting in a total score representing the risk of harm; an AUDIT-C score of ≥3 indicates risky drinking [18,20].

The AUDIT-C was designed primarily to detect risky drinking, alcohol abuse, and addiction in adults, rather than as a sensitive tool for definitively assessing prenatal alcohol exposure. A score of 1–2 on the AUDIT-C represents lower or less frequent alcohol consumption. A score of 3 or greater is defined as risky drinking, while a score above 8 can be interpreted as a significant risk of FASD. In the general population, a threshold of 3 points is more effective, but in prenatal testing, this threshold is often reduced to 1 point to identify any woman who drank even a trace amount of alcohol after conception. Patients often unknowingly underestimate their alcohol consumption due to potential social stigma. For clinical assessment of FASD risk, the key factor is the fact that alcohol exposure occurred at all, not simply whether it exceeded the conventional threshold of “risky drinking.”

The TLFB interview is a validated, calendar-based, retrospective method used to accurately track daily alcohol consumption during pregnancy. It uses memory aids (calendars, special events) to help women recall the exact number of standard drinks consumed, often focusing on pre-conception, specific trimesters, or the 30 days prior to an antenatal visit [21]. 

These methods have many limitations: they are time-consuming and do not effectively capture information about alcohol intake during pregnancy. Other limitations include recall bias, denial due to fear of social disapproval, or providing socially desirable responses regarding abstinence during pregnancy. This leads to an underestimation of maternal alcohol consumption and, consequently, missed diagnosis in many alcohol-exposed children, delaying the provision of developmental and social support. Furthermore, in the case of foster care or adoption, verification cannot be achieved [3]. Therefore, a test that objectively documents fetal alcohol exposure is needed.

Over the past 20 years, attempts have been made to identify candidate biomarkers of PAE that could be useful in predicting potential alcohol-induced damage [22,23].

The aim of this narrative review is to review various tools for assessing PAE, with a particular emphasis on biochemical approaches.

This topic is relevant to contemporary clinical practice with respect to the early diagnosis of FASD and health policy regarding the prevention and/or mitigation of the social consequences of alcohol consumption during pregnancy.

We reviewed selected human studies that focused on the assessment of PAE using a biochemical approach.

We searched the PubMed, MEDLINE, and EBSCO electronic databases dating back to 2000. Only studies involving human participants were considered.

This narrative review provides an overview of selected studies in human cohorts for the detection of intrauterine alcohol exposure.

We summarize the current progress in research on the early diagnosis of FASD, which we hope will provide new insights into early diagnosis in patients prenatally exposed to alcohol. The selection criteria involved selecting relevant studies that linked the confirmation of PAE with laboratory diagnostic tools. Animal studies were excluded. We focused on using biochemical markers in various human matrices to identify prenatal alcohol exposure (PAE) and to facilitate early fetal alcohol spectrum disorders (FASD) diagnosis. Our goal was to scientifically deepen our understanding of the underlying causes of FASD to optimize diagnostic and treatment pathways.

## 2. Assessment of Prenatal Alcohol Exposure by Measuring the Concentration of Fatty Acid Ethyl Esters, Ethyl Sulfate, and Ethyl Glucuronide in Neonatal Meconium

FAEEs are non-oxidative alcohol metabolites produced by fetuses exposed to alcohol. They can be detected in fetal tissues, including the meconium, where they accumulate. Meconium, the first stool passed by the newborn within the first 24–48 h of life, begins to form between the 16th and 18th week of gestation. By measuring ethanol metabolites in this matrix, we can assess PAE during the second and third trimesters of pregnancy, but not the first trimester, as meconium is absent during this period [22].

FAEEs do not cross the placenta and are always of fetal origin. Therefore, their presence directly indicates fetal metabolism following maternal alcohol consumption. They have been analyzed as the sum of several esters (from 4 to 9) or individually (i.e., ethyl laurate (E12:0), ethyl myristate (E14:0), ethyl palmitate (E16:0), ethyl palmitoleate (E16:1), ethyl stearate (E18:0), ethyl oleate (E18:1), ethyl linoleate (E18:2), ethyl linolenate (E18:3), and ethyl arachidonate (E20:4)) [24,25]. Due to the variability between individual FAEEs in meconium samples, it is recommended to sum selected FAEEs to assess PAE.

The cut-off values proposed by various authors for the sum of 4–9 esters that could confirm PAE were set at ≥2 nmol/g (corresponding to 600 ng/g or 0.6 μg/g) [22,23,24,26,27,28,29,30,31]. Other studies have suggested different cut-offs, e.g., Bakdash 2010 proposed a cut-off of >500 ng/g, and other authors have proposed results exceeding the LOQ (limit of quantification) for individual esters to distinguish exposed from unexposed children [32,33]. One study found similar results for the rate of positive FAEEs at cut-offs of 10 and 20 nmol/g. Moreover, the result for the rate of positive FAEEs was similar in newborns whose mothers reported alcohol consumption in the second and third trimesters of pregnancy and those who denied it. A weak association between self-reported alcohol consumption and positive results was observed at a cut-off of >20 nmol/g [34]. The lack of discrimination between exposed and unexposed newborns was likely due to unreliable maternal self-reports. A possible reason could also be the lack of validation of the analytical method in the cited study. In many previous studies, meconium samples collected from infants born to abstinent mothers were used as blanks to confirm the accuracy of the test [25].

In the 2003 study by Chan et al., a positive cut-off value of 2 nmol/g achieved specificity and sensitivity of 98.4% and 100%, respectively [23]. A recent review found relatively good specificity of FAEEs as a biomarker (range 43–100%; median 83%), which was better than their sensitivity (range 4–100%; median 65%) [35]. Finally, based on numerous studies of FAEEs as biomarkers of PAE, a cut-off value for cumulative FAEEs above 2 nmol/g is widely accepted [29,30,36].

Confirmation of PAE using FAEEs has some limitations. Previous studies have indicated the instability of FAEEs at room temperature or following freeze/thaw cycles, but others did not [37]. Detectable concentrations of FAEEs have been found in the meconium of unexposed newborns. For example, olive oil consumption during pregnancy was linked to increased total FAEE concentrations in the meconium of neonates of non-drinking mothers [23,29].

Furthermore, the contamination of meconium with transitional stool because of delayed sample collection can significantly increase FAEE concentrations in this matrix [31,38]. Therefore, it is worth noting that the maternal diet, delayed meconium sampling, neonatal diet prior to sampling and ethanol production by microorganisms inhabiting the neonatal gastrointestinal tract may lead to false positive FAEE results in non-drinking women [31]. Despite these limitations, most authors agree that FAEEs can be a reliable marker of PAE in mothers with an unknown history of alcohol consumption during pregnancy [29,32,33,39]. In studies attempting to establish cut-off points for other biomarkers, the threshold of ≥2 nmol/g has been used to distinguish alcohol-exposed from unexposed children [40].

The possibility of false-positive results has prompted researchers to explore other biomarkers that could complement FAEE analysis [41].

Table 1 presents selected papers regarding PAE determination using FAEEs.

Other markers of alcohol consumption include EtG and EtS, which are water-soluble, non-oxidative metabolites of ethanol. These metabolites can only be measured in biological samples following alcohol consumption. EtG can be detectable in human urine and blood for up to five to six days after heavy alcohol consumption and approximately 24 h after light alcohol use. EtG found in hair may indicate chronic alcohol drinking [44,45].

EtG and EtS were initially used to confirm alcohol consumption in adults and as a screening test to estimate alcohol consumption in pregnant women [46] when abstinence is highly recommended. Urine EtG and EtS testing can help confirm or disprove self-reported alcohol consumption patterns.

The urinary EtG concentration has been reported to be strongly correlated with the amount of alcohol consumed, with a sensitivity of 89.2% and a specificity of 98.8% at a cut-off of >500 ng/mL in the non-pregnant population [47]. Therefore, it is considered a biomarker of recent alcohol consumption, with cut-off levels typically ranging from 100 ng/mL to 500 ng/mL.

Studies indicate that a threshold of 100 ng/mL is more appropriate during pregnancy to detect any alcohol consumption, as even mild alcohol consumption is harmful to the fetus. The higher thresholds of 300–500 ng/mL are more likely to miss moderate or light, occasional alcohol consumption, which is common in early pregnancy [46,48].

The sensitivity of urine EtG is highly dependent on the chosen threshold and the time since consumption, leading to variable results across studies, ranging from 4.9%, 14.8%, and 40.9% to as much as 93% [49,50,51,52].

Urine EtG testing can serve as a valuable tool for monitoring alcohol consumption during pregnancy, as it is suitable for routine testing, verifying suspected alcohol use, or monitoring known consumers. Furthermore, it can reveal moderate to heavy alcohol consumption, as well as low consumption. Given that prenatal alcohol exposure poses a significant global public health challenge, the introduction of a more sensitive urine screening test with a cut-off value of 100 ng/mL is essential to improve detection [53]. This test is quite sensitive and less expensive compared to other methods, but its short detection time remains a limitation—it can reveal alcohol consumption in the last 2–5 days before sample collection. However, its advantage is that it can be performed throughout pregnancy, especially in the first trimester. Detecting alcohol exposure during this vulnerable period for the fetus allows for the implementation of interventions aimed at cessation of drinking by women. It is also useful for monitoring interventions, especially when the risk of fetal damage is high [54]. Due to its short detection time, the test is not suitable for detecting PAE in the absence of a drinking history. Therefore, meconium or hair analysis provides a longer detection window than urine testing, generally during the last few months of pregnancy (around 20 weeks to delivery).

Because EtG and EtS can also be present in the meconium of newborns prenatally exposed to alcohol, they have proven useful as biomarkers of PAE.

Traditional biological matrices such as maternal blood and neonatal urine are routinely used in toxicological screening; however, their short detection windows and invasive collection methods limit their diagnostic utility. In contrast, meconium offers a unique opportunity to retrospectively capture cumulative exposure throughout the second and third trimesters of pregnancy, and it can be collected non-invasively after birth.

EtG is formed by the conjugation of ethanol with glucuronic acid, catalyzed by UDP-glucuronosyltransferase, whereas EtS is the result of the conjugation of ethanol with sulfate by the enzyme sulfotransferase. Unlike FAEEs, EtG crosses the placenta. Because the glucuronidation process in the fetus and newborn is immature and the fetal/neonatal capacity for glucuronidation is limited, this biomarker is largely derived from the mother. EtS is also found in maternal and fetal tissues, including the placenta, umbilical cord and meconium of newborns exposed to alcohol in utero. Studies suggest that EtS is not exclusively of fetal origin and that transplacental transport is also possible [55].

Attempts have been made to establish EtG and EtS thresholds based on maternal self-reported alcohol drinking, but due to significant underreporting, the cut-off values for these biomarkers have often been optimized based on the sum of FAEEs. Cut-offs for EtG from 120 to 440 ng/g or above the LOQ of > 5 ng/g have been proposed [27,29,32,39,56]. Goecke 2014 demonstrated that EtG < 120 ng/g corresponded to alcohol abstinence [39]. Himes et al. concluded that an EtG ≥ 30 ng/g related to maternal self-reported PAE beyond 19 weeks of gestation is optimal for differentiating exposed from unexposed neonates and is superior to the currently known cut-off for FAEEs [29]. Establishing a cut-off for EtS has proven even more difficult, but an EtS above the LOQ, i.e., >1.0–3.0 ng/g, has been proposed (see Table 1).

Some authors reported a correlation between EtG and EtS assessed together, but other researchers have not confirmed this association [27,29]. In a recent study, based on the threshold of FAEEs ≥ 2 nmol/g and maternal self-report, higher cut-off values for EtS and EtG were proposed, i.e., 0.0075 ug/g and 0.05 ug/g (EtS = 8 ng/g and EtG 50 ng/g), respectively. According to this study, both EtS and EtG at the proposed values better correlated with self-reported alcohol consumption during pregnancy than FAEEs. Despite this, a discrepancy between the questionnaire and the biochemical results was noted [36]. Of the 478 women surveyed, 15 women admitted to drinking alcohol once or less a month, and 5 women admitted to drinking alcohol 2 to 4 times a month, representing approximately 4.2% of women [36]. In general, all PAE biomarkers, such as total FAEEs, EtS, and EtG, showed an increase in concentration with increasing and declared frequency of alcohol consumption during pregnancy. Total FAEEs performed worst in this comparison. 

Meconium EtG and EtS measurements may be superior to FAEEs due to their better stability in the meconium and independence from maternal diet. Both cut-off points near the LOD and higher levels were effective in distinguishing alcohol-exposed from unexposed cases. Recent studies have highlighted their advantage over maternal questionnaires in predicting developmental abnormalities [40,42,43].

Furthermore, compared to meconium FAEEs, meconium EtG concentrations correlate better with maternal hair EtG concentrations and with maternal self-assessment [57].

Hair analysis allows for the quantification of maternal drugs and metabolites, with EtG and FAEEs serving as specific biomarkers for ethanol intake [58].

The results of hair ethanol metabolite testing can be influenced by hair melanin content (hair color) and ethnicity. Furthermore, hygiene and cosmetic procedures, such as shampooing, dyeing, the use of cosmetics, and thermal hair treatments, can affect the stability or quantity of metabolites in the hair matrix.

However, the advantage of this matrix is that it accumulates the substances used and, similar to meconium, allows for the assessment of historical alcohol use/exposure, depending on hair length.

Other advantages include the simple and non-invasive sample collection process, the ease of transport, and the ease of storage. However, for some women, collecting samples may be culturally inappropriate.

Assuming hair length increases by approximately 1 cm per month, a hair length of 9 cm will allow for exposure assessment throughout pregnancy, including the first trimester. However, to avoid detecting pre-pregnancy consumption, some authors recommend sampling 6 cm, which reflects exposure in the second and third trimesters, similar to meconium. Hair testing appears to be superior to meconium testing in terms of collection and storage (at room temperature). However, as with meconium, it requires sample preparation and the use of chromatographic methods.

Studies examining the accuracy of hair EtG in pregnant women [27,59] and non-pregnant populations [60,61] have yielded mixed results. A study conducted in a non-pregnant population of chronically alcohol-dependent individuals demonstrated an overall sensitivity of 96% and a specificity of 99% for hair EtG [62]. However, studies among pregnant women regarding the sensitivity and specificity of this biomarker compared to self-assessments have also yielded inconsistent results.

In a study of 103 pregnant Swedish women [59], a positive hair EtG test identified more women (15.5%) with some level of alcohol exposure than the AUDIT questionnaire (8.7%). In an Italian study, all 99 samples collected from mother–infant pairs were negative for hair EtG and hair fatty acid ethyl esters, whereas 82.8% of meconium samples were positive for EtG and 22.2% for FAEEs [27]. Perhaps the method of confirming PAE using medical records rather than a thorough personal interview may have limited the validation of hair EtG as a reliable biomarker.

According to studies, hair EtG measurements do not appear to be suitable for differentiating low-to-moderate drinking over a 3-month period versus complete abstinence [60,63]. After considering the washout of possible alcohol metabolites due to cosmetic procedures, the sensitivity of the test is reduced.

Meconium collection is non-invasive; a minor drawback is the need for storage at −20 °C, but this can be easily overcome. The assessment of the accuracy of exposure detection and cut-off points is widely studied and relatively well understood. The FRAMES and FRANCES studies, which included follow-up of children from birth to school age, demonstrated that meconium EtG levels ≥ 10 ng/g correlated with neurobehavioral disorders [32,39,42,43]. At the same time, no correlation was found with self-reported alcohol consumption, and mothers of children above the cut-off point indicating chronic, heavy alcohol consumption denied drinking, which would make it difficult to correctly identify these children [64]. A Canadian study found that the positive rate of meconium FAEEs was 10 times higher compared to self-reported data [31]. For ethical reasons, accurate verification of maternal alcohol consumption is impossible, which poses a key challenge in determining the sensitivity and specificity of meconium biomarkers in human studies. Whereas in animal models, when the amount of ethanol administered to the pregnant female was known, a sensitivity and specificity of 93.3% for cumulative FAEEs were demonstrated [65,66]. Therefore, detecting PAE using objective methods is important and clinically useful, despite known limitations that must be considered when interpreting the results. After all, the goal of PAE assessment is to identify a group of children and their families early to provide them with early neurodevelopmental interventions. This is especially important when a drinking history is unknown, as in the case of adoption/foster care, or when the mother denies it.

Although the meconium stores information about exposure to toxicants from the second and even third trimesters of pregnancy, this information is still useful because PAE is harmful at every stage of pregnancy and translates into neurodeficiencies, the detection of which is important for providing developmental and social support to the child and their family. For the first trimester, easily available and cheaper tests, e.g., urine EtG, can be implemented.

Examples of studies on ethanol metabolites in urine and hair are presented in Table 2.

## 3. Markers of Growth Failure and Neurobehavioral Disorders in Children Exposed to Alcohol

In recent years, researchers have focused on the pathomechanism of the alcohol-induced changes that influence the pregnancy course and outcome. These findings provide a better understanding of how alcohol alters the transplacental allocation of resources between mother and fetus. Understanding the effects of alcohol on the activity of neurotransmitters, growth factors and hormones contributes to the translation of these modifications into the occurrence of dysmorphic features, alcohol-related birth defects and neurodevelopmental disorders in offspring prenatally exposed to alcohol.

The placenta provides interaction between the pregnant mother and the fetus. It not only mediates the supply of nutrients and oxygen but also ensures the maintenance of hormonal and metabolic balance in the mother and her offspring, ensuring the proper utilization of maternal resources. In particular, the insulin/Insulin-like Growth Factor (IGF) system, i.e., insulin, IGF1, and IGF2, modulated by IGF-binding proteins (IGFBP-1 to IGFBP-6) and specific receptors (IGF1R, IGF2R), the insulin receptor (INSR) and the IGF1R–INSR hybrid receptor, plays a key role in the regulation of fetal and placental growth and development [70,71]. The activation of the IGF system directly affects target tissues, influencing metabolism, mitogenesis, survival, and differentiation of many cell types [71]. IGFs exert direct and indirect effects on placental growth, promoting its growth and capacity, exchange surface area, transport functions and hormone secretion. IGFs regulate glucose and lipid metabolism and amino acid transport across the placenta, making them important determinants of fetal and postnatal growth and development. IGF1 also regulates glucose metabolism in the developing brain, promoting brain cell proliferation and differentiation, stimulating neurogenesis and myelination and preventing apoptosis, thus appearing to be essential for neurological outcomes. IGF1 is the major linear growth factor in early childhood, acting independently of pituitary growth hormone. Mice deficient in IGF1 had significantly lower birth weights than normal, and growth retardation persisted into adulthood [71,72,73]. Therefore, dysregulation of insulin and IGFs profoundly affects both the placenta and the fetus. These effects may persist beyond the fetal period and manifest in childhood, influencing individuals’ growth and development. An association between the maternal serum levels of total IGF1, free IGF1, IGFBPs, and fetal or neonatal growth has been reported. Decreased maternal IGF1 concentrations have been observed in fetuses with fetal growth restriction, while increased IGF1 concentrations were noted in pregnant women with diabetes and in cases of Beckwith–Wiedeman syndrome [74,75]. Alcohol-induced changes in the concentration and function of IGF1 and IGF2 may significantly impact fetal programming and contribute to growth restriction. Heavy maternal drinking has been shown to inhibit placental and fetal growth [73,76,77]. Animal studies suggest dysregulation of the IGF1 signaling pathway following alcohol exposure, but this regulation is insufficiently studied in humans, and the reported results are inconsistent [73,77,78]. A study by Aros et al. confirmed reduced anthropometric measurements in alcohol-exposed newborns along with significantly reduced IGF2 and slightly reduced IGF1 levels. Over time, a catch-up growth was observed, accompanied by an increase in IGF1 and IGF2 levels. At 3–5 years of age, these levels were significantly higher than those in unexposed children [79]. In contrast, another study in school-age children showed significantly reduced levels of IGF1 and IGF2 in individuals with FASD and PAE compared to the control group [80]. Furthermore, decreased IGF2 was associated with a smaller head circumference and decreased total intelligence and cognitive skills in alcohol-exposed children [80]. This is consistent with other observational studies, which found that growth failure correlated with poor cognitive ability [81,82]. A recent study has also shown reduced IGF1 levels at birth, although without an impact on neonatal size. However, a delayed effect of PAE on growth was observed. The harmful effects of ethanol persisted beyond the prenatal period, contributing to adverse growth and nutritional statuses, which manifested around 6 months of age as poorer growth outcomes compared to unexposed patients [76].

Alcohol-induced disruption of IGF signaling, beginning in utero, may contribute to pre- and postnatal growth retardation and neurobehavioral disorders. Growth factor testing, along with the assessment of growth patterns from birth through early childhood, may be a useful biomarker of PAE. Furthermore, it may predict the severity of neurodevelopmental damage in exposed children.

The results of human studies on IGF regulation are presented in Table 3.

Several animal studies indicate that alcohol consumption disrupts vascular endothelial growth factor (VEGF)-mediated angiogenesis, leading to impaired placental vascularization. This disruption of vascular development is implicated in placental insufficiency and subsequent fetal growth restriction [81,82,83].

Human studies have also demonstrated alcohol-induced changes in the vascularization at the maternal–fetal interface, which limit nutrient and oxygen delivery to the fetus [84].

Alterations in the gene network responsible for angiogenesis have been demonstrated using RNA sequencing of the human placenta. This was reflected in the reduced birth weight in infants exposed to alcohol prenatally, suggesting a link between altered gene pathways and adverse fetal/neonatal growth and neurodevelopmental outcomes [85].

Furthermore, changes in the expression of placental proteins mediating angiogenesis have been demonstrated, as well as an imbalance between pro- and anti-inflammatory molecules such as tumor necrosis factor-alpha (TNF-α) and interleukin-13 (IL-13) [86]. Increased levels of proinflammatory cytokines, such as IL-1β, IL-6, and TNF-α, have been observed in infants born to mothers who chronically consumed alcohol during pregnancy but not among mothers who drank moderately [87]. However, recent studies have shown that even light to moderate alcohol consumption disrupts the balance of these molecules, leading to increased levels of primarily proinflammatory cytokines [88]. Studies assessing changes in angiogenesis-related and inflammatory processes are presented in Table 4 and Table 5.

## 4. The Role of Neurotrophins in Brain Development

Neurotrophins are important factors that play a crucial role in normal brain development and mediate synaptic plasticity in adulthood. These include nerve growth factor (NGF), brain-derived neurotrophic factor (BDNF), neurotrophin-3 (NT-3), and NT-4/5. They act through the activation of the tropomyosin-related kinase (Trk) receptors: NGF binds to TrkA, and BDNF binds to TrkB.

Their biological role is to stimulate several intracellular signaling pathways, thereby promoting the survival, growth, differentiation, and maintenance of both neuronal and non-neuronal cells. During central nervous system development, alcohol-induced alterations in the balance of NGF and BDNF and their receptors negatively impact the development of neuronal connections, consequently impairing learning and memory skills.

A number of studies in animal models showed that prenatal alcohol exposure affects NGF and BDNF production in different brain areas, mainly in the hippocampus and cortex. The link was found between NGF and BDNF disruptions, TrkA and TrkB expression and altered cognition and emotional behavior [89,90,91,92,93].

Studies show that reduced levels of BDNF and NGF in the hippocampus and cerebral cortex lead to impaired spatial learning, reduced synaptic plasticity (long-term potentiation), and memory impairment. NGF/BDNF dysfunction is associated with widespread cell death (apoptosis) and impaired neurogenesis, particularly during critical periods of hippocampal and cortical development. Changes in neurotrophin expression are not transient but persist throughout life, contributing to long-term behavioral deficits and depressive behavior in offspring of heavy drinkers [89,90,93,94,95]. Another study demonstrated that ethanol exposure during synaptogenesis induces massive apoptotic neurodegeneration in the developing mammalian brain [96]. These findings highlight a critical mechanism underlying fetal alcohol syndrome.

## 5. Inflammation as a Consequence of PAE

The harmful effects of alcohol on the fetus manifest through disruption of various cellular processes. Recent, extensive research indicates that neuroinflammation may be a key factor in the development of neurodegenerative diseases such as Alzheimer’s and Parkinson’s diseases [97,98,99]. It also appears that neuroinflammation may play a significant role in the development of FASD symptoms. Neuroinflammation refers to the intricate response of the CNS to specific insults, such as infections, hypoxia, and internal and external toxins, including alcohol. Immune cells and molecules are key players in fetal brain growth and maturation. During fetal life, astrocytes and microglia serve as both builders and defenders of the developing CNS. They guide neurogenesis and promote neuronal proliferation, cell migration and differentiation. They support the formation and maturation of synapses, regulate the reuptake and recycling of neurotransmitters and play a role in the formation and regulation the blood–brain barrier. The primary role of microglia is to act as immune system cells. By regulating chemokine activity, they contribute to proper brain development and function, improving neural networks through synaptic pruning. The interaction between astrocytes and microglia leads to a controlled inflammatory response aimed at repairing or limiting damage [99,100]. In some circumstances, their response to insults has both beneficial and detrimental effects on the developing brain. Studies have demonstrated that early exposure to ethanol leads to long-term, impaired microglial/astrocyte activation and the increased production of proinflammatory mediators, including cytokines (IL-1β, IL-6, and TNF-α) and other inflammatory molecules such as COX-2 and iNOS. This is accompanied by the decreased production of anti-inflammatory cytokine such as IL-10 [101]. Alcohol exposure also results in the altered production of chemokines such as CCL2, known as monocyte chemoattractant protein-1, CXCL1, and CXCL16, with a concomitant decrease in the ani-inflammatory and neuroprotective chemokine CX3CL1 [102]. The imbalance of these molecules has been linked to alcohol-induced neuroinflammation in the developing CNS. Studies in mice have shown that disordering the CX3CL1–CX3CR1 signaling pathway disrupts normal synaptogenesis and synaptic pruning [103,104]. Proinflammatory CCL2 and neutrophil chemokine-12a (CXCL12), in addition to their primary function of regulating the inflow of inflammatory cells to the brain, also influence the migration and growth of developing neurons. Through exacerbation of the inflammatory process, they may inhibit normal neuronal growth, which results in a reduction in neuronal and glial cell populations [105,106]. Alcohol-induced inflammatory changes in brain structure and function subsequently manifest as cognitive and behavioral deficits observed in patients with FASD.

The study analyzing the serum protein profile in a cohort of children diagnosed with FASD showed that the proinflammatory state may persist after birth into childhood. FASD-diagnosed patients demonstrated increased expression of proinflammatory molecules, namely, IFNγ, IL-1β, IL-6, and CX3CL1, accompanied by decreased levels of CXCL16 and IL-10 [101]. Studies in mice have shown that maintaining a balance between CX3CL1 and CXCL16 has a neuroprotective effect. Acting together, they modulate the interaction between neurons, microglia, and astrocytes and regulate the release of CCL2 from astrocytes, contributing to the mitigation of brain damage. Maintaining a balance in the CX3CL1-CXCL16-CCL2 signaling pathway is necessary for these chemokines to act synergistically. In the absence of CXCL16, the neuroprotective effect of CX3CL1 is attenuated [103]. Studies from Ukraine have also demonstrated the importance of maintaining a balance between pro- and anti-inflammatory factors on fetal outcomes. An alcohol-induced maternal cytokine imbalance, particularly an elevated TNF-α to IL-10 ratio, increased the risk of neurological disorders in the offspring [107]. Increased levels of IL-6, CRP, IFNɣ, TNF-β, and TNF-α, as well as IL-10, were found in the serum of pregnant women consuming alcohol, which correlated with delayed neurological development in their children. Moreover, children of mothers with the highest cytokine levels were more likely to have a thin vermillion border [108]. This may support the conception that neuroinflammation translates into both physical impairments, including the typical morphological features of FASD, as well as cognitive and behavioral disorders.

According to literature, molecules such as IL-10, IFNγ, IL-1β, CCL2, CX3CL1, and CXCL16 are the most important for the pathophysiology of FASD. While some studies have shown overexpression of IL-10 [108], its decreased levels are more frequently observed in response to alcohol exposure [101,109]. Reduced IL-10 levels are associated with a high possibility of FASD diagnosis. Similarly, elevated levels of IFNγ, IL-1β, and CCL2 demonstrate a strong positive association with predicting FASD. The ability to measure these biomarkers in blood opens a novel diagnostic approach. Laboratory, minimally invasive assays can facilitate diagnosis regardless of the time of sample collection, even without a known history of fetal alcohol exposure. Moreover, reducing excessive inflammation and chemokine levels may offer neuroprotection, making an understanding of these pro- and anti-inflammatory molecules key to new therapeutic strategies.

Examples of studies assessing the effect of PAE on the inflammatory process are presented in Table 5.

**Table 5:** Effect of PAE on inflammatory process.

Author PAE Confirmation	Cohort Characteristics	Inflammatory Markers	Comments
Ahluwalia 2000 [87] Cord blood samples from neonates born to mothers who drank alcohol moderately to heavily. Washington, University Hospital	PAE group, n = 16 including heavy alcohol users, n = 8, moderate alcohol users, n = 8 Unexposed, n = 8	↑ IL-6, IL-1β, TNF-α, IL-1 α	Significant increases in IL-1β, IL-6, and TNF-α in newborns born to mothers who chronically drank alcohol during pregnancy. No differences between abstainers and moderate drinking mothers.
Maxwell 2024 [88] Cord blood samples and peripheral blood of light-to-moderate drinking mothers University of New Mexico clinic providing care for substance abuse and addiction disorders	PAE group, n = 8, Unexposed, n = 10	↑ IFNɣ, IL-12p70, IL-1β, IL-2, IL-6, TNF-α, IL-4, ↑ IL-13, IL-4, IL-8	Changes in the levels of proinflammatory and anti-inflammatory cytokines in cord blood samples from newborns of mothers who drank light to moderate amounts of alcohol
Ramos-Triguero 2025 [101] Spanish and adopted from EEC children PAE confirmation: self-report—none, Physical and neurological assessment by Hoyme 2016 [5] Matrix examined: Child’s serum	PAE group—none, FAS diagnosed, n = 60,	Increased ↑ IFNγ, CX3CL1, IL-1β, ↑ IL-6, CCL2, Lower IL-10, CXCL16	-
	Unexposed, n = 30		
Bodnar 2018 [108] Ukraine, Rivne and Khmelnytsky Region, 2007–2012 Matrix examined: Maternal blood samples collected during the second and third trimesters of pregnancy	PAE group, n = 57, Unexposed, n = 95	↑ IFNɣ, IL-10, TNF-β, TNF-α, CRP ↑ IL-15, IL-10	Increase in cytokine and chemokine levels in maternal blood correlated with neurodevelopmental delay and facial dysmorphism in exposed children
Sowel 2018 [107] Ukraine, Rivne and Khmelnytsky Region, 2007–2012 Matrix examined: Maternal blood samples collected during the second and third trimesters of pregnancy	Moderate/heavy alcohol exposed, n = 149, Low/non-exposed, n = 92	↑ TNF-α/IL-10 ratio; ↑ IL-6/IL-10 ratio	FASD diagnosis: Children’s neurological assessment based on the Bayley Scales of Infant Development II, Mental Development Index (MDI), or Psychomotor Development Index (PDI), Outcomes: Negatively associated with neurological outcomes in exposed children

EEC—Eastern Europe Countries.

## 6. Molecular Approach: Why Do Some Children Prenatally Exposed to Alcohol Not Exhibit Severe Symptoms of FAS?

The teratogenic effects of alcohol on the placenta and fetus depend, among other factors, on the individual genetic background. Furthermore, the way PAE impacts the FAS phenotype depends on epigenetic modifications in alcohol-exposed individuals. The primary mechanism underlying the altered epigenetic programming is differential methylation of certain gene loci, which influences gene expression without affecting the genomic sequence. Several human studies have identified a number of differentially methylated sites and regions within genes and pathways that have been identified as potentially characteristic for FASD [85,110,111,112,113]. Genome-wide analysis of DNA methylation patterns using buccal swabs from children diagnosed with FASD revealed altered DNA methylation patterns in this group compared to typically developed controls [114]. Differential methylation of genes playing key role in epigenetic processes has been found to influence neuronal differentiation. Some of these genes have been identified as associated with neurodevelopmental disorders, reduced IQ and alterations in attention-related processes [40,114]. Methylation changes following PAE occur primarily in placental and brain tissues. It has been suggested that because buccal epithelial cells originate from the same germ layer as neural cells, they can act as surrogates and be used to assess and predict neurobiological changes, which could provide a novel strategy for the diagnosis of FASD [114].

Among the many factors that significantly influence placental and fetal growth, the imprinted gene complex, i.e., IGF2/H19 locus, plays a crucial role. The locus is regulated by allele-specific DNA methylation at the H19 imprinting control region (H19 ICR), the H19 promoter region (H19 DMR) and in the IGF2 differentially methylated regions (DMR0, DMR1, and DMR2). Proper function of this locus, and thus proper embryonic and fetal development, requires both maternal and paternal copies, where the paternal allele expresses IGF2 and the maternal—H19. Many studies suggest that paternal IGF2 expression is primarily growth promoting, whereas the H19 RNA gene product, i.e., non-coding RNA, is maternally expressed and growth-limiting [111,115]. It has been shown that hypomethylation of the H19 ICR receptor in humans results in reduced IGF2 expression and biallelic H19 expression, leading to fetal growth restriction (FGR) or other severe prenatal and postnatal growth retardation syndromes, i.e., Russell–Silver syndrome [116]. In contrast, hypermethylation of the H19 ICR receptor leads to overexpression of IGF2 and reduced H19 expression, resulting in excessive fetal growth, as in Beckwith–Wiedemann syndrome [117]. Alcohol exposure during fetal life alters gene expression in the insulin–IGF signaling pathway in a parent-of-origin manner. A Finnish human study supports the thesis that the effect of PAE on the regulation at the IGF2/H19 locus is genotype-specific and related to parental origin [118]. In alcohol-exposed placentas of a specific genotype, a single nucleotide polymorphism in the paternal allele was associated with a significant reduction in the DNA methylation level at the H19 ICR and decreased IGF2 expression. In placentas of other genotypes, a relative increase in H19 expression compared to IGF2 was observed, which highlighted an imbalance in the regulation of these genes following PAE. Furthermore, this specific genotype correlated with a significantly smaller head circumference in affected newborns. Neonates with a different genotype had head circumferences similar to the population average, despite prenatal alcohol exposure [118]. This may confirm the results of previous observational studies, which did not show the reduced head circumference in alcohol-exposed children [119,120]. Mutations in the CTCF gene, encoding the protein regulating the IGF2/H19 region, have been described in some cases of mild intellectual disability, certain congenital defects (heart defects, cleft palate, microcephaly), and growth restriction [121]. PAE causes a similar phenotype through a shared mechanism involving the epigenetic dysregulation of these genetic loci.

In the Cape Town cohort, PAE resulted in decreased anthropometric measurements at birth, including body weight, length and head circumference, compared to unexposed individuals. Growth retardation persisted throughout infancy and early childhood. Reduced placental weight was also observed. Unlike the Swedish study, this was accompanied by increased IGF2 expression in exposed placentas [77]. This may seem inconsistent with previous studies suggesting that IGF2 is growth promoting. However, the authors of this study did not investigate the parent-of-origin allele expression, which could have biased the results. Furthermore, the teratogenic effect of alcohol on growth could have resulted from further changes in allele-independent expression or the action of other factors [77]. Similarly, increased IGF2 activity following PAE has been observed in human trophoblast cultures and animal models [122]. The results of these studies provide evidence that imprinted genes are involved in fetal and postnatal growth and development. Further research is needed to identify candidate genes that could be markers of PAE and be used to predict alcohol-induced impairments in growth and neurodevelopment.

The results of human studies on genetic testing in PAE are presented in Table 6.

## 7. Whether Causal Treatment Is Possible

Until now, it was thought that alcohol-induced changes in the developing brain were irreversible and that potential improvements in the functioning of exposed individuals could be achieved through early social and behavioral interventions to mitigate neurobehavioral damage. Using anti-inflammatory medications and nutraceuticals to reduce inflammation may prove to be a promising approach. In vitro and in vivo studies have demonstrated a positive effect of exogenous CXCL16 administration on reducing brain damage [103]. In human studies, the administration of epigallocatechin gallate (EGCG), a catechin found in green tea, effectively restored IL-10 levels without affecting CXCL16 levels. The therapeutic effect likely resulted from downregulation of other proinflammatory factors [101,123]. Further research on suppressing inflammation is necessary and feasible. This therapy may be effective in limiting the effects of PAE.

## 8. Directions of Changes

The deleterious effects of alcohol on the pregnancy course and outcomes are mediated by multiple mechanisms. Epigenetic changes in gene regulation through differential DNA methylation patterns, occurring primarily in placental and brain tissue, negatively impact feto-placental growth and development. Research suggests that mechanisms leading to fetal and postnatal growth impairment and neurodevelopmental disorders share a common mechanism [124]. Altered methylation at the IGF and H19 genes inhibits cell proliferation, migration and differentiation, influencing fetal and postnatal growth and neurobiology [40,125]. Alcohol-induced neuroinflammation through an imbalance between pro- and anti-inflammatory molecules also plays a significant role in the occurrence of cognitive and behavioral deficits in FASD patients [101].

Given the diverse effects of alcohol on pregnancy and the fetus, as well as its impact on numerous aspects of fetal development, it is difficult to find a test that would allow for a definitive diagnosis of PAE. Nevertheless, advances in understanding the effects of alcohol on the activity of neurotransmitters, growth factors, and gene expression contribute to the diagnosis of PAE.

Introducing laboratory assays for proinflammatory molecules in the diagnosis of FASD appears to be a promising and accessible diagnostic method, especially since some of these cytokines, such as IL-10, IL-6, IL-1β, TNF-α, are already in widespread laboratory and clinical use. Growth factor testing, along with the evaluation of growth patterns from birth through early childhood, may be a useful diagnostic tool for assessing PAE, as IGF1 testing is also available [76,80]. Furthermore, this approach may help predict the severity of neurodevelopmental damage in exposed children. Identifying genes that could be potential markers of PAE appears to be a promising approach. Moreover, this assay may prove useful during pregnancy and be used to predict alcohol-induced growth and neurodevelopmental disorders. Therefore, it can be considered a preventative measure, although further research is needed to standardize this tool.

While numerous diagnostic tools for PAE detection have shown potential, an optimal method has yet to be determined. Researchers agree that the test should effectively differentiate alcohol-exposed from unexposed individuals, have a low false positive rate, and the matrix should be easy to collect, store, and analyze. To date, the determination of alcohol metabolites in meconium is the most studied biochemical method, with established procedures for collection, storage, and analysis of samples, as well as established cut-off values [29,36,56]. However, it has certain limitations: dependence on the maternal and neonatal diet and a limited sampling time. Therefore, combining this method with clinical assessment and other laboratory assays, such as blood tests, may offer an effective and feasible approach. In the context of alcohol-induced altered gene expression that translates into growth disturbances, combining biochemical methods with anthropometric measurements at birth and the subsequent assessment of growth trajectory also appears to be a feasible diagnostic approach [126].

However, it should be noted that while the molecular approach is promising, it is still the subject of intensive research and requires replication and validation before it becomes standard and reliable in clinical practice. Combining these biomarkers with artificial intelligence (machine learning) models could improve their accuracy and create a reliable diagnostic tool.

Research into the effectiveness of other tests shows promise, such as testing for inflammation markers in the blood. Because blood sampling is minimally invasive, introducing these tests could facilitate diagnosis regardless of the time of sampling or known history of fetal alcohol exposure.

Further research is needed to identify laboratory tests that could be used across different settings to assess alcohol exposure and predict alcohol-induced growth and neurodevelopmental disorders.

## Figures and Tables

**Table 1:** Assessment of prenatal alcohol exposure by measuring the concentration of fatty acid ethyl esters, ethyl sulfate, and ethyl glucuronide in neonatal meconium: FAEEs, EtG, EtS.

Author Study Timeframe	Country Region Population	FAEEs LOD LOQ Cut-Off	EtG LOD LOQ Cut-Off	EtS LOD LOQ Cut-Off	Self-Reported Prevalence of PAE	Meconium Testing Prevalence of PAE/Comments
Chan 2003 [23]	Canada, Toronto, n = 102, Israel, Jerusalem, n = 104	Cumulative 7 FAEEs ≥ 2 nmol/g	-	-	Toronto n = 15, Jerusalem n = 2, Six confirmed alcohol use mothers	Negative correlation between ethyl palmitate and birth weight, as well as head circumference
Gareri 2008 [22] January 2004–February 2005	Canada, Ontario, Grey Bruce Region, Primary perinatal care setting, n = 682	Cumulative FAEEs ≥ 2 nmol/g n = 17	Not applicable	Not applicable	Questionnaire 0.5%	2.5%
Goh 2010 [24] August 2006–July 2007	Canada, Ontario, Grey Bruce Region, High-risk perinatal setting, n = 50	Individual ethyl esters: LOD range 15–50 ng/g, Cumulative 4 FAEEs ≥ 2 nmol/g	-	-	Parkyn questionnaire 1.0%	PAE by cumulative FAEEs: 15 (30%)
Bakdash 2010 [32] FRAMES study 2005–2007	Germany, Frankonia, Nuremberg, n = 602	Cumulative 4 FAEEs > 500 ng/g n = 43	596 cases LOD = 10 ng/g, LOQ = 12 ng/g Cut-off > 274 ng/g	-	Questionnaire 1%	PAE by FAEEs: 7.1% (43) PAE by EtG LOD > 10 ng/g: 16.3% (97) PAE by EtG > 274 ng/g 5.5% (33) Combined positivity for two biomarkers (FAEEs + EtG) 5.5%
Pichini 2009 [26]	Reggio Emilia, Italy, n = 96 Barcelona, Spain, n = 81	Cumulative FAEEs ≥ 2 nmol/g (0.6 ug/g equivalent ≥ 600 ng/g)	LOQ > 5 ng/g Cut-off not established	LOQ > 1 ng/g Cut-off not established	Questionnaire 5%	PAE by EtG: 81.5% Italy, 95.5% Spain PAE by EtS: 46.9% Italy, 51.9% Spain PAE by FAEEs: 8% Italy, 42% Spain
Morini 2010a [27]	Reggio Emilia, Italy, n = 49, Barcelona, Spain, n = 50	Cumulative 7 FAEEs ≥ 2 nmol/g	LOD = 0.007 nmol/g LOQ—0.022 nmol/g EtG range: 0.031–6.53 nmol/g, Proposed cut-off = 1.5 nmol/g (≥330 ng/g) or >2.0 nmol/g (440 ng/g)	LOD = 0.002 nmol/g (0.26 ng/g); LOQ—0.008 nmol/g (1.06 ng/g) EtS range: 0.008–0.039 nmol/g. Proposed Cut-off = 0.012 nmol/g (1.6 ng/g) or >0.020 nmol/g (2.6 ng/g)	Data from medical records	PAE by EtG LOQ: 82.8% (n = 82) both groups PAE by EtS LOQ: 19.2% (n = 19) both groups PAE by FAEEs: 10.4% Italy, 34% Spain, both groups: 22.2% (n = 22) Significant correlation between EtG and EtS
Morini 2010b [28]	Initially 185 Reggio Emilia, Italy, n = 50 Barcelona, Spain, n = 60	Cumulative 7 FAEEs ≥ 2 nmol/g	EtG range: 0.022–1.87 nmol/g, Cut-off ≥ 2 nmol/g (440 ng/g)	EtS range: 0.008–0.030 nmol/g Cut-off not established	Questionnaire	-
Kwak 2014 [34]	Republic of Korea, Seul, n = 294	Cumulative 9 FAEEs > 2 nmol/g; FAEEs > 10 nmol/g FAEEs > 20 nmol/g	-	-	Questionnaire n = 112 (38.1%)	PAE by FAEEs no significant differences between abstainers and alcohol drinkers; no correlation between PAE and anthropometric parameters at birth
Himes 2015 [29]	US Northern Plains, n = Cape Town, South Africa, n = 107	Cumulative 7 FAEEs ≥ 2 nmol/g LOQ 25–50 ng/g	LOQ 5 ng/g Cut-off ≥ 30 ng/g	LOQ 2.5 ng/g	Timeline Follow-Back interview No alcohol 33 during pregnancy, PAE beyond 19 GA 54% (n = 58)	PAE by FAEEs 23.4% (n = 25) PAE by EtG ≥ 30 ng/g 42% (n = 45), Correlation between self-reported PAE and PAE by EtG ≥ 30 ng/g. Weak correlation between EtG and individual or cumulative FAEEs, No correlation between EtG and EtS
Lee H 2018 [30] April–August 2016	Korea, Uijeongbu, N = 157	Cumulative 9 FAEEs > 2 nmol/g LOD range 0.01–0.08 nmol/g, LOQ range 0.02–0.27 nmol/g	-	-	Questionnaire and interview, 8.9% during each trimester; 2.5% during second to third trimesters	PAE by FAEEs 2.5%. The higher the FAEE concentration, the greater the risk of reduced head circumference and length <10th percentile
Delano 2019 [31] 2008–2011	Canada, multicenter study (10 cities), N = 1315	Cumulative 4 FAEEs > 2 nmol/g	-	-	Questionnaire, 0.24% heavy drinking, 32% low–moderate drinking	PAE by FAEEs 1.16–2.4% Depending on the timing of meconium sampling. The later the sampling, the greater the risk of false positive results
Bakhireva 2019 [33] April 2013–November 2016	US Navajo Birth Cohort, N = 333	Individual ethyl esters (9) LOD range 10–25 ng/g LOQ 15–50 ng/g	LOQ > 5 ng/g	LOQ > 3 ng/g	AUDIT-C ≥ 3 [risky drinking] 12.5%	PAE by FAEEs range 1.2–6.9% PAE by EtG LOQ > 5 ng/g; 5.1% PAE by EtS LOQ > 3 ng/g; 7.8% Combined positivity for two meconium biomarkers 5.4%
Wiergowski 2026 [36] June 2019–April 2020	Gdańsk, Poland, N = 478	Cumulative 9 FAEEs ≥ 2 nmol/g	Cut-off > 50 ng/g (0.05 ug/g)	Cut-off > 8 ng/g (0.0075 ug/g)	Questionnaire 4.2%	Better correlations between both biomarkers (EtG and EtS) and self-reported data than for FAEEs, PAE estimated at 3%
Goecke 2014 [39] 2005–2007 FRAMES study population 986	Germany, Frankonia, Nuremberg, N = 557	Individual ethyl esters (5) LOD/LOQ N.A.	≥120 ng/g	-	Questionnaire 21.2%	PAE by EtG 14% (26) PAE by EtG inverted correlation with self-reported drinking
Eichler 2018 [42] FRAMES study: Meconium samples at birth FRANCES I (2012–2015) neuropsychological assessment	Germany, Frankonia, Nuremberg, School-age children, n = 44 exposed n = 44 unexposed	-	1st cut-off ≥10 ng/g; 2nd cut-off ≥154 ng/h	-	Questionnaire	At both cut-off points–cognitive deficits, cut-off point ≥ 154 ng/g—lower IQ, ADHD-related behaviors, no correlation with maternal self-reported data
Roetner 2023 [43] FRAMES study: Meconium samples at birth FRANCES II (2019–2021) neuropsychological assessment	Germany, Frankonia, Nuremberg, School-age children, n = 96: n = 25 exposed n = 71 unexposed	-	≥10 ng/g	-	Questionnaire	EtG ≥ 10 ng/g neuropsychological deficits, no correlation with maternal self-reported data

ADHD—attention-deficit/hyperactivity disorder, FAEEs—fatty acid ethyl esters, EtG—ethyl glucuronide, EtS—ethyl sulfate, FRAMES—Franconian Maternal Health Evaluation Study, FRANCES—Franconian Cognition and Emotion Study, LOD—limit of detection.

**Table 2:** Assessment of prenatal alcohol exposure by measuring the concentration of fatty acid ethyl esters, ethyl sulfate, and ethyl glucuronide in various specimens.

Author Matrix Examined	Country Region Population Sample Size	FAEE Cut-Off	EtG Cut-Off	EtS LOD LOQ Cut-Off	Selfe-Reported Prevalence of PAE	Comments
Wurst 2008 [59] Maternal hair 6 cm	Uppsala University Hospital, pregnant women admitted for routine second trimester ultrasound screening, n = 2008, urine samples, n = 103	For FAEE in hair, LOD 0.7 pg/mg, LOQ 2.3 pg/mg	Social drinkers hEtG ≥7 to <25 pg/mg, heavy drinkers hair EtG ≥ 25 pg/mg urine EtG 1 positive sample	urine EtS LOD 0.05 mg/L, LOQ 0.11 mg/L 0 positive samples	AUDIT > 5 N = Nine women (8.7%)	Urine samples tested, only 1 was positive for EtG. In hair testing, 19 cases were tested Positive, 16 for EtG, 3 for FAEEs. PAE by AUDIT combined with biomarkers—n = 26 (25.2%)
Joya X 2016 [57] Maternal hair 9 cm, Neonatal meconium	Barcelona, Spain, n = 80	Meconium Cumulative seven FAEE ≥ 2 nmol/g	Meconium EtG ≥ 30 ng/g n = 50 Hair EtG Abstainers < 7 pg/mg n = 22 (27.5%), >7 pg/mg > N = 57 (72.5%)		Questionnaire 19 women (23.8%)	The best significant correlations (*p* < 0.0001) were found between EtG concentration in the proximal 0–3 cm and 3–6 cm hair shaft segments corresponding to the last two pregnancy trimesters and EtG in neonatal meconium, Hair EtG > 11 pg/mg better correlated with meconium EtG ≥30 n/g
Gomez-Roig M. 2018 [67] Maternal hair 9 cm at labor In single aliquot	Barcelona, Spain, n = 153 February to May 2016 Maternity center	-	Cut-off > 7 pg/mg, Repetitive moderate drinking EtG 7–30 pg/mg, heavy drinking > 30 pg/mg		Questionnaire N = 4 (2.6%)	Abstainers by hair EtG n = 54 (35.3%) Moderate drinkers by hair EtG: n = 96 (62.7%) Heavy drinkers by hair EtG: n = 3 (2%)
Morini 2010a [27] Maternal and neonatal hair Forensic Science International	Reggio Emilia, Italy, n = 49; Barcelona, Spain, n = 50	Cumulative 7 FAEE LOD 10–40 pg/mg LOQ 40–120 pg/mg for different FAEEs Maternal hair n = 26	LOD 2 pg/mL LOQ 3 pg/mL Maternal hair n = 99, Neonatal hair n = 35		Data from medical records	By hair testing—No chronic ethanol abusers
Corrales-Gutierrez [68] Toxics	Sevilla, Public hospital, Spain, n = 425		Maternal hair EtG Abstainers < 5 pg/mg, Consumers >5 pg/mg		Interview cohort n = 425, Hair testing cohort n = 252	PAE by questionnaire 20.7%, PAE by hair testing 20.2%
Bakhireva 2014 [48]	University of New Mexico clinic providing care for those with substance abuse and addiction disorders; PAE n = 28, Controls n = 32		Maternal urine mid-gestation GA 22, and at delivery urine EtG ≥ 25 ng/mL	Maternal urine mid-gestation and at delivery urine EtS ≥ 7 ng/mL	AUDIT-C ≥ 3	Low sensitivity and high specificity
Gutierrez 2015 [63] Hair 6 cm	University of New Mexico clinic providing care for those with substance abuse and addiction disorders		uEtG ≥ 25 ng/mL, [48]	uEtS ≥ 7 ng/mL	Questionnaire 0.5% TLFB	2.5%
Ferraguti 2017 [49]	Sapienza University Hospital in Roma, n = 70 including n = 46 alcohol drinking, n = 24 abstainers		Maternal urine EtG > 100 ng/mL commercial enzyme immunoassays	-	Questionnaire N = 46 (65.7%) alcohol drinkers, 24 abstainers	PAE by urine EtG 34.28% No correlation between questionnaire and urine EtG testing
Ferraguti 2020 [69]	Sapienza University Hospital in Roma, n = 44		Maternal urine >100 ng/mL = occasional drinking	-	Questionnaire	PAE by EtG n = 15 (34.09%) Outcome: the presence of physical features of FASD on prenatal ultrasound screening in the second trimester more frequent in EtG-positive women
Ceci 2021 [52] Period 2016–2020	Ambulatory of Gynaecology and Obstetrics, Sapienza University Hospital in Roma, n = 309		Maternal urine EtG ≥100 ng/mL commercial enzyme immunoassays; EtG/CreU ratio > 103 ng/g	-	Questionnaires N = 46 (65.7%) alcohol drinkers, 24 abstainers	PAE by Questionnaires 65.7% PAE by urine EtG—11–14.6%, PAE by EtG/CreU ratio—20–25.6% No correlation between questionnaire and urine EtG testing
Jolma 2023 [44]	Finland: Helsinki and Lahti, five settings at various levels of perinatal care, including specialist antenatal clinic for pregnant women with problematic substance use, n = 505		Urine rapid EtG strips with cut-off > 300 ng/mL, Quantitative assay: Cut-off >100 and <300 ng/mL			PAE by rapid urine test: clear positive n = 5 (1%), clear negative n = 443 (87.7%), uncertain n = 57 (11.3%); PAE by quantitative testing of uncertain samples and randomly selected negative samples in rapid test n = 38 (7.5%)
McCormick C.A. 2026 [46]	Ireland, multicenter study, one urban and two semirural maternal units, n = 1053		Urine Enzyme immunoassay: Cut-off > 500 ng/mL			Positive samples n = 4 (0.38%)

**Table 3:** The impact of prenatal alcohol exposure on growth factor levels and growth outcomes in children.

Author, PAE Confirmation, Cohort Characteristics	Cohort Characteristics	Biomarkers of PAE	Anthropometry		Comments
			At Birth	Follow-Up	
Aros 2011 [79] Chile, Children from birth to 5 years old, PAE confirmation: self-report, Matrix examined: Child’s serum	PAE group, n = 69	↓ IGF1 at 1 month of age, ↑ IGF1 from 6 months of age and increased with age, ↓ IGF2 at 1 month of age, ↑ IGF2 from 6 month of age and increased with age, IGFBP3 no differences	SGA ↓ Weight ↓ Length	Up 5 years of age Catch-up growth, No differences between groups	Decreased correlation between IGF1 and growth parameters
	FAS diagnosed—none				
	Unexposed, n = 83				Strong correlation between IGF1 and growth parameters
Andreu-Fernandez 2019 [80] Spanish and adopted from EEC children aged 8–12 years, PAE confirmation: self-report, Physical and neurological assessment, PAE by meconium FAEEs ≥ 2 nmol/g, Matrix examined: Child’s serum	PAE group, n = 33	↓ IGF1 (gender-dependent manner—in females) ↓ IGF2	-	-	
	FAS diagnosed, n = 86	↓ IGF1 from 6–8 years of age ↓ IGF2	-	8–12 years of age ↓ HC ↓ Length ↓ Weight	Correlation between decreased IGFs and growth and neurocognitive impairment
	Unexposed, n = 31				
Barry 2024 [76] Philippines, Children from birth to two years old, PAE confirmation: self-report, PEth ≥ 8 ng/dL in maternal DBS, Matrix examined: Cord blood, Child’s serum	PAE group, n = 93 FAS diagnosed—none	↓ IGF1 at birth and 6 months IGF1 12 m no differences	No differences in HC, length, weight	Up to 2 years HC—No differences ↓ Length ↓ Weight ↓ MUAC	Present correlation between IGF1 and growth parameters in both groups
	Unexposed, n = 203				

DBS—dried blood spot, HC—head circumference, IGF—insulin-like growth factor, MUAC—mid-upper arm circumference, PAE—prenatal alcohol exposure, SGA—small for gestational age.

**Table 4:** Angiogenesis-related processes.

Author, PAE Confirmation	Cohort Characteristics	Matrix Examined, Results	Anthropometry		Comments
			At Birth	Follow-Up	
Deyssenroth 2024 [85] Cape Town, South Africa, Newborns/children up to 5 y PAE confirmation: self-report, FASD diagnosis—clinical assessment according to Hoyme guidelines (2005)	PAE group, n = 33 (8 children with FASD diagnosis), Unexposed, n = 39	Placenta Placental RNA sequencing, gene expression analysis Altered gene expression in genes related to angiogenesis	↓ HC ↓ Length ↓ Weight	↓ HC ↓ Length ↓ Weight	↓ Placental weight
Holbrook 2019 [86], University of New Mexico clinic providing care for substance abuse and addiction disorders PAE confirmation: test AUDIT score ≥ 8, TLFB	PAE group, n = 13 Control, n = 13	Placenta Altered expression of angiogenesis-related proteins and cytokines ↑ TNF-α ↑ IL-13 ↓ VEGFR2 ↓ VEGFR1 ↓ CCM-3	-	-	-

Angiogenesis-related proteins: CCM-3—Cavernous Malformation Protein 3, VEGFR1 and 2—vascular endothelial growth factor receptors, Pro- and anti-inflammatory molecules: IL—interleukin, TNF-α—tumor necrosis factor alpha.

**Table 6:** Molecular assessment.

Author, PAE Confirmation	Cohort Characteristics	Matrix Examined, Results	Anthropometry		Comments
			At Birth	Follow-Up	
Portales-Casamar 2016 [114] Canada, children age 11 y +/− 3.3 years PAE confirmation: Physical and neurological assessment by Chudley 2005 [6]	FASD or PAE confirmed group, n = 110	Children’s buccal swabs—Buccal Epithelial Cells (BECs) Differential DNA methylation pattern in FASD children: Hypomethylation of H19 imprinted gene—↑H19 expression	-	-	Possible association of FASD differentially methylated loci with neurodevelopmental disorders in FASD cases
	Unexposed, n = 96 with typical development				
Marjonen 2017 [118] Finland, Newborns, PAE confirmation: test AUDIT	PAE group, n = 39	Placenta ↑ H19 expression in a parent-of-origin manner (hypomethylation at H19 ICR in the paternal allele) ↑ H19 expression > IGF2 expression in all alcohol-exposed placentas ↓ IGF2 expression in genotype-specific manner	↓ HC ↓ Length ↓ Weight	-	↓ HC related to genotype-specific alcohol-induced alteration in methylation level of IGF2/H19 ICR
	Unexposed, n = 100				
Carter 2018 [77] Cape Town, South Africa, Newborns/children up to 5 years old PAE confirmation: self-report, FASD diagnosis—clinical assessment according to Hoyme guidelines (2005) [4]	PAE group, n = 34 (8 children with FASD diagnosis)	Placenta ↑ IGF2 gene expression	↓ HC ↓ Length ↓ Weight	↓ HC ↓ Length ↓ Weight	↓ Placental weight
	Unexposed, n = 31				
Frey 2018 [40] FRAMES/FRANCES cohort, Meconium samples at birth, Long-term effects of PAE assessment	PAE by EtG ≥ 30 ng/g, n = 32; unexposed, n = 125	Cognitive development (IQ test, EEG), Buccal swab—Whole-genome methylation patterns of buccal cell DNA, Gene-specific analysis			PAE group: lower IQ, ADHD-related behaviors, 193 genes differentially methylated

ADHD—attention-deficit/hyperactivity disorder, EEG—electroencephalogram, HC—head circumference, IGF—insulin-like growth factor, PAE—prenatal alcohol exposure.

## Data Availability

No new data were created or analyzed in this study. Data sharing is not applicable to this article.

## Abbreviations

The following abbreviations are used in this manuscript:

ADHD	Attention deficit/hyperactivity disorder
ARBD	Alcohol-related birth defect
ARND	Alcohol-related neurodevelopmental disorder
AUDIT-C	Alcohol Use Disorders Identification Test—consumption subset
CCL2	Monocyte chemoattractant protein-1
CNS	Central nervous system
COX	Cyclooxygenase
CRP	C-reactive protein
DMR	Differentially methylated region
DNA	Deoxyribonucleic acid
EGCG	Epigallocatechin gallate
EtG	Ethyl glucuronide
EtS	Ethyl sulfate
E 12:0	Ethyl laurate
E 14:0	Ethyl myristate
E 16:0	Ethyl palmitate
E 16:1	Ethyl palmitoleate
E 18:0	Ethyl stearate
E 18:1	Ethyl oleate
E 18:2	Ethyl linoleate
E 18:3	Ethyl linolenate
E 20:4	Ethyl arachidonate
FAEEs	Fatty acid ethyl esters
FASD	Fetal alcohol spectrum disorder
FGR	Fetal growth restriction
FRAMES	Franconian Maternal Health Evaluation Study
FRANCES	Franconian Cognition and Emotion Study
H19 ICR	H19 imprinting control region
IGF	Insulin-like growth factor
IGFBP	IGF-binding protein
IGFR	IGF Receptor
IGFR–INSR	IGF Receptor–insulin receptor
INSR	Insulin receptor
IL	Interleukin
IFNγ	Interferon gamma
iNO	Nitric oxide
IQR	Interquartile range
LOD	Limit of detection
LOQ	Limit of quantification
LLOQ	Lower limit of quantification
IOM	Institute of Medicine
pFAS	partial fetal alcohol syndrome
PAE	Prenatal alcohol exposure
RNA	Ribonucleic acid
SGA	Small for gestational age
TLFB	Timeline Follow-Back
TNF-α	Tumor necrosis factor α

## References

[1] IOM (Institute of Medicine—IOM of the National Academy of Science) (1996). Executive Summary. Fetal Alcohol Syndrome: Diagnosis, Epidemiology, Prevention, and Treatment.

[2] Wozniak J.R., Riley E.P., Charness M.E. (2019). Diagnosis, epidemiology, assessment, pathophysiology, and management of fetal alcohol spectrum disorders. Lancet Neurol..

[3] Popova S., Charness M.E., Burd L., Crawford A., Hoyme H.E., Mukherjee R.A.S., Riley E.P., Elliott E.J. (2023). Fetal alcohol spectrum disorders. Nat. Rev. Dis. Primers.

[4] Hoyme H.E., May P.A., Kalberg W.O., Kodituwakku P., Gossage J.P., Trujillo P.M., Buckley D.G., Miller J.H., Aragon A.S., Khaole N. (2005). A practical clinical approach to diagnosis of fetal alcohol spectrum disorders: Clarification of the 1996 institute of medicine criteria. Pediatrics.

[5] Hoyme H.E., Kalberg W.O., Elliott A.J., Blankenship J., Buckley D., Marais A.S., Manning M.A., Robinson L.K., Adam M.P., Abdul-Rahman O. (2016). Updated clinical guidelines for diagnosing fetal alcohol spectrum disorders. Pediatrics.

[6] Chudley A.E., Conry J., Cook J.L., Loock C., Rosales T., LeBlanc N. (2005). Fetal alcohol spectrum disorder: Canadian guidelines for diagnosis. CMAJ.

[7] Cook J.L., Green C.R., Lilley C.M., Anderson S.M., Baldwin M.E., Chudley A.E., Conry J.L., LeBlanc N., Loock C.A., Lutke J. (2016). Fetal alcohol spectrum disorder: A guideline for diagnosis across the lifespan. CMAJ.

[8] Watkins R.E., Elliott E.J., Wilkins A., Mutch R.C., Fitzpatrick J.P., Payne J.M., O’Leary C.M., Jones H.M., Latimer J., Hayes L. (2013). Recommendations from a consensus development workshop on the diagnosis of fetal alcohol spectrum disorders in Australia. BMC Pediatr..

[9] Landgraf M.N., Nothacker M., Kopp I.B., Heinen F. (2013). The Diagnosis of Fetal Alcohol syndrome. Dtsch. Arztebl. Int..

[10] Okulicz-Kozaryn K., Maryniak A., Borkowska M., Śmigiel R., Dylag K.A. (2021). Diagnosis of Fetal Alcohol Spectrum Disorders (FASDs): Guidelines of Interdisciplinary Group of Polish Professionals. Int. J. Environ. Res. Public Health.

[11] May P.A., Chambers C.D., Kalberg W.O., Zellner J., Feldman H., Buckley D., Kopald D., Hasken J.M., Xu R., Honerkamp-Smith G. (2018). Prevalence of Fetal Alcohol Spectrum Disorders in 4 US Communities. JAMA.

[12] Kalberg W.O., May P.A., Buckley D., Hasken J.M., Marais A.S., de Vries M.M., Bezuidenhout H., Manning M.A., Robinson L.K., Adam M.P. (2019). Early-life predictors of fetal alcohol spectrum disorders. Pediatrics.

[13] Patel T., Narula S., Naderzad E., Early D., Nandhagopal T. (2022). Fetal Alcohol Spectrum Disorder in a Newborn. Cureus.

[14] Jolma L.M., Koivu-Jolma M., Sarajuuri A., Torkki P., Autti-Rämö I., Sätilä H. (2023). Children with FASD—Evolving Patterns of Developmental Problems and Intervention Costs in Ages 0 through 16 in Finland. Children.

[15] Oh S.S., Kuang I., Jeong H., Song J.Y., Ren B., Moon J.Y., Park E.C., Kawachi I. (2023). Predicting Fetal Alcohol Spectrum Disorders Using Machine Learning Techniques: Multisite Retrospective Cohort Study. J. Med. Internet Res..

[16] Mattson S.N., Jones K.L., Chockalingam G., Wozniak J.R., Hyland M.T., Courchesne-Krak N.S., del Campo M., Riley E.P. (2023). Validation of the FASD-Tree as a screening tool for fetal alcohol spectrum disorders. Alcohol Clin. Exp. Res..

[17] Bakhireva L.N., Leeman L., Roberts M., Rodriguez D.E., Jacobson S.W. (2021). You Didn’t Drink During Pregnancy, Did You?. Alcohol Clin. Exp. Res..

[18] Freeman J., Condon C., Hamilton S., Mutch R.C., Bower C., Watkins R.E. (2019). Challenges in Accurately Assessing Prenatal Alcohol Exposure in a Study of Fetal Alcohol Spectrum Disorder in a Youth Detention Center. Alcohol Clin. Exp. Res..

[19] Lim Y.H., Watkins R.E., Jones H., Kippin N.R., Finlay-Jones A. (2022). Fetal alcohol spectrum disorders screening tools: A systematic review. Res. Dev. Disabil..

[20] Marini C., Northover N.S., Gold N.D., Rogers U.K., O’Donnell K.C., Tofighi B., Ross S., Bogenschutz M.P. (2023). A Systematic Approach to Standardizing Drinking Outcomes from Timeline Followback Data. Subst. Abus. Res. Treat..

[21] Baker T., Haynes R.L., Paterson D.S., Broadbelt K.G., Markianos K., Holm I.A., Boyd T., Roberts D., Goldstein R.G., Stein H. (2017). A modified Timeline Followback assessment to capture alcohol exposure in pregnant women: Application in the Safe Passage Study. Alcohol.

[22] Gareri J., Lynn H., Handley M., Rao C., Koren G. (2008). Prevalence of fetal ethanol exposure in a regional population-based sample by meconium analysis of fatty acid ethyl esters. Ther. Drug Monit..

[23] Chan D., Bar-Oz B., Pellerin B., Paciorek C., Klein J., Kapur B., Farine D., Koren G. (2003). Population baseline of meconium fatty acid ethyl esters among infants of nondrinking women in Jerusalem and Toronto. Ther. Drug Monit..

[24] Goh Y.I., Hutson J., Lum L., Roukema H., Gareri J., Lynn H., Koren G. (2010). Rates of fetal alcohol exposure among newborns in a high-risk obstetric unit. Alcohol.

[25] Woźniak M.K., Banaszkiewicz L., Aszyk J., Wiergowski M., Jańczewska I., Wierzba J., Kot-Wasik A., Biziuk M. (2021). Development and validation of a method for the simultaneous analysis of fatty acid ethyl esters, ethyl sulfate and ethyl glucuronide in neonatal meconium: Application in two cases of alcohol consumption during pregnancy. Anal. Bioanal. Chem..

[26] Pichini S., Morini L., Marchei E., Palmi I., Rotolo M.C., Vagnarelli F., Garcia-Algar O., Vall O., Zuccaro P. (2009). Ethylglucuronide and ethylsulfate in meconium to assess gestational ethanol exposure: Preliminary results in two Mediterranean cohorts. Can. J. Clin. Pharmacol..

[27] Morini L., Marchei E., Vagnarelli F., Garcia Algar O., Groppi A., Mastrobattista L., Pichini S. (2010). Ethyl glucuronide and ethyl sulfate in meconium and hair-potential biomarkers of intrauterine exposure to ethanol. Forensic Sci. Int..

[28] Morini L., Groppi A., Marchei E., Vagnarelli F., Algar O.G., Zuccaro P., Pichini S. (2010). Population Baseline of Meconium Ethyl Glucuronide and Ethyl Sulfate Concentrations in Newborns of Nondrinking Women in 2 Mediterranean Cohorts. Ther. Drug Monit..

[29] Himes S.K., Dukes K.A., Tripp T., Petersen J.M., Raffo C., Burd L., Odendaal H., Elliott A.J., Hereld D., Signore C. (2015). Clinical sensitivity and specificity of meconium fatty acid ethyl ester, ethyl glucuronide, and ethyl sulfate for detecting maternal drinking during pregnancy. Clin. Chem..

[30] Lee H.S., Kim Y.H., Kwak H.S., Han J.Y., Jo S.J., Lee H.K. (2018). Association of fatty acid ethyl esters in meconium of neonates with growth deficits at birth: A prospective, single-centre cohort study. J. Korean Med. Sci..

[31] Delano K., Koren G., Zack M., Kapur B.M. (2019). Prevalence of Fetal Alcohol Exposure by Analysis of Meconium Fatty Acid Ethyl Esters; A National Canadian Study. Sci. Rep..

[32] Bakdash A., Burger P., Goecke T.W., Fasching P.A., Reulbach U., Bleich S., Hastedt M., Rothe M., Beckmann M.W., Pragst F. (2010). Quantification of fatty acid ethyl esters (FAEE) and ethyl glucuronide (EtG) in meconium from newborns for detection of alcohol abuse in a maternal health evaluation study. Anal. Bioanal. Chem..

[33] Bakhireva L.N., Kane M.A., Bearer C.F., Bautista A., Jones J.W., Garrison L., Begay M.G., Ozechowski T., Lewis J. (2019). Prenatal alcohol exposure prevalence as measured by direct ethanol metabolites in meconium in a Native American tribe of the southwest. Birth Defects Res..

[34] Kwak H.S., Han J.Y., Choi J.S., Ahn H.K., Kwak D.W., Lee Y.K., Koh S.Y., Jeong G.U., Velázquez-Armenta E.Y., Nava-Ocampo A.A. (2014). Dose-response and time-response analysis of total fatty acid ethyl esters in meconium as a biomarker of prenatal alcohol exposure. Prenat. Diagn..

[35] Kable J.A., Jones K.L. (2023). Identifying Prenatal Alcohol Exposure and Children Affected by It: A Review of Biomarkers and Screening Tools. Alcohol Res..

[36] Wiergowski M., Jańczewska I., Wierzba J., Cichoń-Kotek M., Woźniak M.K., Kot-Wasik A., Biziuk M., Anand J.S., Schetz D.B., Glińska M. (2026). Estimation of Prenatal Alcohol Exposure: Comparison of Retrospective Survey and Measurement of Fatty Acid Ethyl Esters, Ethyl Sulfate, and Ethyl Glucuronide Concentrations in Neonatal Meconium. Toxics.

[37] Himes S.K., Concheiro M., Scheidweiler K.B., Huestis M.A. (2014). Validation of a novel method to identify in utero ethanol exposure: Simultaneous meconium extraction of fatty acid ethyl esters, ethyl glucuronide, and ethyl sulfate followed by LC-MS/MS quantification. Anal. Bioanal. Chem..

[38] Zelner I., Hutson J.R., Kapur B.M., Feig D.S., Koren G. (2012). False-Positive Meconium Test Results for Fatty Acid Ethyl Esters Secondary to Delayed Sample Collection. Alcohol Clin. Exp. Res..

[39] Goecke T.W., Burger P., Fasching P.A., Bakdash A., Engel A., Häberle L., Voigt F., Faschingbauer F., Raabe E., Maass N. (2014). Meconium Indicators of Maternal Alcohol Abuse during Pregnancy and Association with Patient Characteristics. BioMed Res. Int..

[40] Frey S., Eichler A., Stonawski V., Kriebel J., Wahl S., Gallati S., Goecke T.W., Fasching P.A., Beckmann M.W., Kratz O. (2018). Prenatal Alcohol Exposure Is Associated with Adverse Cognitive Effects and Distinct Whole-Genome DNA Methylation Patterns in Primary School Children. Front. Behav. Neurosci..

[41] Suttie M., Kable J., Mahnke A.H., Bandoli G. (2024). Machine learning approaches to the identification of children affected by prenatal alcohol exposure: A narrative review. Alcohol Clin. Exp. Res..

[42] Eichler A., Hudler L., Grunitz J., Grimm J., Raabe E., Goecke T.W., Fasching P.A., Beckmann M.W., Kratz O., Moll G.H. (2018). Effects of prenatal alcohol consumption on cognitive development and ADHD-related behaviour in primary-school age: A multilevel study based on meconium ethyl glucuronide. J. Child Psychol. Psychiatry.

[43] Roetner J., van Doren J., Maschke J., Kulke L., Pontones C., Fasching P.A., Beckmann M.W., Lenz B., Kratz O., Moll G.H. (2023). Effects of prenatal alcohol exposition on cognitive outcomes in childhood and youth: A longitudinal analysis based on meconium ethyl glucuronide. Eur. Arch. Psychiatry Clin. Neurosci..

[44] Bager H., Christensen L.P., Husby S., Bjerregaard L. (2017). Biomarkers for the Detection of Prenatal Alcohol Exposure: A Review. Alcohol Clin. Exp. Res..

[45] Ghosh S., Jain R., Rao R., Mishra A.K., Jhanjee S. (2023). Does ethyl glucuronide in hair correlate with alcohol consumption? A comparative study with other traditional biomarkers among individuals with alcohol dependence syndrome. Alcohol.

[46] Jolma M., Koivu-Jolma M., Niemelä O., Autti-Rämö I., Kahila H. (2023). Rapid urine screening for ethyl glucuronide from pregnant women as a tool for detecting prenatal alcohol exposure. BMC Pregnancy Childbirth.

[47] McDonell M.G., Srebnik D., Angelo F., Sugar A.M., Howell D., Rainey C., Roll J., Short R., Ries R. (2011). Evaluation of ethyl glucuronide immunoassay urinalysis in five alcohol-dependent outpatients. Am. J. Addict..

[48] McCormick C.A., Mealy G., Donaghy C., Gordon P., Dunn E., Carroll P., Rodriguez-Herrera A., Cusnaider C.M., Ebbitt W., McCormick P.A. (2026). Urinary alcohol and ethyl glucuronide as a screening tool for alcohol use in pregnancy: A multicenter prospective study. Acta Obstet. Gynecol. Scand..

[49] Gutierrez H.L., Hund L., Shrestha S., Rayburn W.F., Leeman L., Savage D.D., Bakhireva L.N. (2015). Ethylglucuronide in maternal hair as a biomarker of prenatal alcohol exposure. Alcohol.

[50] Bakhireva L.N., Leeman L., Savich R.D., Cano S., Gutierrez H., Savage D.D., Rayburn W.F. (2014). The Validity of Phosphatidylethanol in Dried Blood Spots of Newborns for the Identification of Prenatal Alcohol Exposure. Alcohol. Clin. Exp. Res..

[51] Ferraguti G., Ciolli P., Carito V., Battagliese G., Mancinelli R., Ciafrè S., Tirassa P., Ciccarelli R., Cipriani A., Messina M.P. (2017). Ethylglucuronide in the urine as a marker of alcohol consumption during pregnancy: Comparison with four alcohol screening questionnaires. Toxicol. Lett..

[52] Lowe J.M., McDonell M.G., Leickly E., Angelo F.A., Vilardaga R., McPherson S., Srebnik D., Roll J., Ries R.K. (2015). Determining ethyl glucuronide cutoffs when detecting self-reported alcohol use in addiction treatment patients. Alcohol Clin. Exp. Res..

[53] Bakhireva L.N., Savage D.D. (2011). Focus on: Biomarkers of fetal alcohol exposure and fetal alcohol effects. Alcohol Res. Health.

[54] Ceci F.M., Fiore M., Agostinelli E., Tahara T., Greco A., Ralli M., Polimeni A., Lucarelli M., Colletti R., Angeloni A. (2021). Urinary Ethyl Glucuronide for the Assessment of Alcohol Consumption During Pregnancy: Comparison between Biochemical Data and Screening Questionnaires. Curr. Med. Chem..

[55] Jennifer H., Lendoiro E., de Castro A., Gónzalez-Colmenero E., Concheiro-Guisan A., Peñas-Silva P., Macias-Cortiña M., Cruz-Landeira A., López-Rivadulla M., Concheiro-Guisan M. (2019). Detection of in utero ethanol exposure via ethyl glucuronide and ethyl sulfate analysis in umbilical cord and placenta. Forensic Toxicol..

[56] Pichini S., Marchei E., Vagnarelli F., Tarani L., Raimondi F., Maffucci R., Sacher B., Bisceglia M., Rapisardi G., Elicio M.R. (2012). Assessment of prenatal exposure to ethanol by meconium analysis: Results of an Italian multicenter study. Alcohol Clin. Exp. Res..

[57] Joya X., Marchei E., Salat-Batlle J., García-Algar O., Calvaresi V., Pacifici R., Pichini S. (2016). Fetal exposure to ethanol: Relationship between ethyl glucuronide in maternal hair during pregnancy and ethyl glucuronide in neonatal meconium. Clin. Chem. Lab. Med..

[58] del Campo M., Jones K.L. (2017). A review of the physical features of the fetal alcohol spectrum disorders. Eur. J. Med. Genet..

[59] Wurst F.M., Kelso E., Weinmann W., Pragst F., Yegles M., Sundström Poromaa I. (2008). Measurement of direct ethanol metabolites suggests higher rate of alcohol use among pregnant women than found with the AUDIT-a pilot study in a population-based sample of Swedish women. Am. J. Obstet. Gynecol..

[60] Kronstrand R., Brinkhagen L., Nyström F.H. (2012). Ethyl glucuronide in human hair after daily consumption of 16 or 32 g of ethanol for 3 months. Forensic Sci. Int..

[61] Politi L., Morini L., Leone F., Polettini A. (2006). Ethyl glucuronide in hair: Is it a reliable marker of chronic high levels of alcohol consumption?. Addiction.

[62] Boscolo-Berto R., Favretto D., Cecchetto G., Vincenti M., Kronstrand R., Ferrara S.D., Viel G. (2014). Sensitivity and Specificity of EtG in Hair as a Marker of Chronic Excessive Drinking. Ther. Drug Monit..

[63] Vermeulen L., van Nuijs A.L.N., Crunelle C.L., Jacobs W., Neels H. (2022). Ethyl glucuronide and alcohol abstinence: A correlation study in hair and fingernails to establish a cut-off value in fingernails for teetotalers. Forensic Sci. Int..

[64] Eichler A., Grunitz J., Grimm J., Walz L., Raabe E., Goecke T.W., Beckmann M.W., Kratz O., Heinrich H., Moll G.H. (2016). Did you drink alcohol during pregnancy? Inaccuracy and discontinuity of women’s self-reports: On the way to establish meconium ethyl glucuronide (EtG) as a biomarker for alcohol consumption during pregnancy. Alcohol.

[65] Littner Y., Cudd T.A., O’Riordan M.A., Cwik A., Bearer C.F. (2008). Elevated fatty acid ethyl esters in meconium of sheep fetuses exposed in utero to ethanol—A new animal model. Pediatr. Res..

[66] Zelner I. (2013). Screening for Prenatal Alcohol Exposure Using Meconium Fatty Acid Ethyl Esters as Biomarkers. Ph.D. Thesis.

[67] Gomez-Roig M.D., Marchei E., Sabra S., Busardò F.P., Mastrobattista L., Pichini S., Gratacós E., Garcia-Algar O. (2018). Maternal hair testing to disclose self-misreporting in drinking and smoking behavior during pregnancy. Alcohol.

[68] Corrales-Gutierrez I., Gomez-Baya D., Leon-Larios F., Medero-Canela R., Marchei E., Mendoza-Berjano R., García-Algar Ó. (2023). Alcohol Consumption Assessed by a Biomarker and Self-Reported Drinking in a Sample of Pregnant Women in the South of Europe: A Comparative Study. Toxics.

[69] Ferraguti G., Merlino L., Battagliese G., Piccioni M.G., Barbaro G., Carito V., Messina M.P., Scalese B., Coriale G., Fiore M. (2020). Fetus morphology changes by second-trimester ultrasound in pregnant women drinking alcohol. Addict. Biol..

[70] Smith S.M. (2025). Nonconceptus Mechanisms of Prenatal Alcohol Exposure That Disrupt Embryo-Fetal Development: An Integrative View. Alcohol Res..

[71] Martín-Estal I., Castilla-Cortázar I., Castorena-Torres F. (2021). The Placenta as a Target for Alcohol During Pregnancy: The Close Relation with IGFs Signaling Pathway. Rev. Physiol. Biochem. Pharmacol..

[72] Hellström A., Ley D., Hansen-Pupp I., Hallberg B., Ramenghi L.A., Löfqvist C., Smith L.E., Hård A.L. (2016). Role of Insulinlike Growth Factor 1 in Fetal Development and in the Early Postnatal Life of Premature Infants. Am. J. Perinatol..

[73] Martín-Estal I., Fajardo-Ramírez O.R., Bermúdez De León M., Zertuche-Mery C., Rodríguez-Mendoza D., Gómez-Álvarez P., Galindo-Rangel M., Leal López A., Castilla-Cortázar I., Castorena-Torres F. (2024). Ethanol consumption during gestation promotes placental alterations in IGF-1 deficient mouse placentas. F1000Research.

[74] Wen J., Zhao Y., Xian R., Fan X. (2026). Correlation between umbilical cord concentration of the growth hormone-IGF axis and small for gestational age: A single-center retrospective, observational study. Int. J. Gynaecol. Obstet..

[75] Su J., Huang X., Meng S., Wang S. (2025). Investigating Serum and Placental Levels of IGF-1 and IGF-1R in Preeclampsia Patients and Their Clinical Implications. Int. J. Womens Health.

[76] Barry C.V., Chrysanthopoulou S.A., Tallo V., Jarilla B., Vargas Z., McDonald E., Gundogan F., Friedman J.F. (2024). The Impact of Prenatal Alcohol Exposure on Longitudinal Growth, Nutritional Status, and Insulin-Like Growth Factor 1 in Early Childhood in Leyte, the Philippines. J. Pediatr..

[77] Carter R.C., Chen J., Li Q., Deyssenroth M., Dodge N.C., Wainwright H.C., Molteno C.D., Meintjes E.M., Jacobson J.L., Jacobson S.W. (2018). Alcohol-Related Alterations in Placental Imprinted Gene Expression in Humans Mediate Effects of Prenatal Alcohol Exposure on Postnatal Growth. Alcohol Clin. Exp. Res..

[78] Pielage M., el Marroun H., Odendaal H.J., Willemsen S.P., Hillegers M.H.J., Steegers E.A.P., Rousian M. (2023). Alcohol exposure before and during pregnancy is associated with reduced fetal growth: The Safe Passage Study. BMC Med..

[79] Aros S., Mills J.L., Iñiguez G., Avila A., Conley M.R., Troendle J., Cox C., Cassorla F. (2011). Effects of prenatal ethanol exposure on postnatal growth and the insulin-like growth factor axis. Horm. Res. Paediatr..

[80] Andreu-Fernández V., Bastons-Compta A., Navarro-Tapia E., Sailer S., Garcia-Algar O. (2019). Serum concentrations of IGF-I/IGF-II as biomarkers of alcohol damage during foetal development and diagnostic markers of Foetal Alcohol Syndrome. Sci. Rep..

[81] Haghighi Poodeh S., Salonurmi T., Nagy I., Koivunen P., Vuoristo J., Räsänen J., Sormunen R., Vainio S., Savolainen M.J. (2012). Alcohol-induced premature permeability in mouse placenta-yolk sac barriers in vivo. Placenta.

[82] Wang G., Zhong S., Zhang S.Y., Ma Z.L., Chen J.L., Lu W.H., Cheng X., Chuai M., Lee K.K., Lu D.X. (2016). Angiogenesis is repressed by ethanol exposure during chick embryonic development. J. Appl. Toxicol..

[83] Gualdoni G.S., Ventureira M.R., Coll T.A., Palomino W.A., Barbeito C.G., Cebral E. (2021). Perigestational alcohol consumption induces altered early placentation and organogenic embryo growth restriction by disruption of trophoblast angiogenic factors. Reprod. Biomed. Online.

[84] Carter R.C., Jacobson J.L., Molteno C.D., Dodge N.C., Meintjes E.M., Jacobson S.W. (2016). Fetal alcohol growth restriction and cognitive impairment. Pediatrics.

[85] Deyssenroth M.A., Williams R.P., Lesseur C., Jacobson S.W., Jacobson J.L., Cheng H., Bose P., Li Q., Wainwright H., Meintjes E.M. (2024). Prenatal alcohol exposure is associated with changes in placental gene co-expression networks. Sci. Rep..

[86] Holbrook B.D., Davies S., Cano S., Shrestha S., Jantzie L.L., Rayburn W.F., Bakhireva L.N., Savage D.D. (2019). The association between prenatal alcohol exposure and protein expression in human placenta. Birth Defects Res..

[87] Ahluwalia B., Wesley B., Adeyiga O., Smith D.M., Da-Silva A., Rajguru S. (2000). Alcohol modulates cytokine secretion and synthesis in human fetus: An in vivo and in vitro study. Alcohol.

[88] Maxwell J.R., Noor S., Pavlik N., Rodriguez D.E., Enriquez Marquez L., DiDomenico J., Blossom S.J., Bakhireva L.N. (2024). Moderate Prenatal Alcohol Exposure Increases Toll-like Receptor Activity in Umbilical Cord Blood at Birth: A Pilot Study. Int. J. Mol. Sci..

[89] Carito V., Ceccanti M., Ferraguti G., Coccurello R., Ciafrè S., Tirassa P., Fiore M. (2017). NGF and BDNF Alterations by Prenatal Alcohol Exposure. Curr. Neuropharmacol..

[90] Boschen K.E., Klintsova A.Y. (2016). Neurotrophins in the Brain: Interaction With Alcohol Exposure During Development. Vitam. Horm..

[91] Fiore M., Laviola G., Aloe L., di Fausto V., Mancinelli R., Ceccanti M. (2009). Early exposure to ethanol but not red wine at the same alcohol concentration induces behavioral and brain neurotrophin alterations in young and adult mice. NeuroToxicology.

[92] Zeng X., Cai Y., Wu M., Chen H., Sun M., Yang H. (2024). An overview of current advances in perinatal alcohol exposure and pathogenesis of fetal alcohol spectrum disorders. J. Neurodev. Disord..

[93] Aloe L. (2006). Alcohol intake during prenatal life affects neuroimmune mediators and brain neurogenesis. Ann. Ist. Super. Sanita.

[94] Caldwell K.K., Sheema S., Paz R.D., Samudio-Ruiz S.L., Laughlin M.H., Spence N.E., Roehlk M.J., Alcon S.N., Allan A.M. (2008). Fetal Alcohol Spectrum Disorder-associated depression: Evidence for reductions in the levels of brain-derived neurotrophic factor in a mouse model. Pharmacol. Biochem. Behav..

[95] Alhowail A. (2022). Mechanisms Underlying Cognitive Impairment Induced by Prenatal Alcohol Exposure. Brain Sci..

[96] Ikonomidou C., Bittigau P., Ishimaru M.J., Wozniak D.F., Koch C., Genz K., Price M.T., Stefovska V., Hörster F., Tenkova T. (2000). Ethanol-induced apoptotic neurodegeneration and fetal alcohol syndrome. Science.

[97] Domingues C., da Cruz E., Silva O.A.B., Henriques A.G. (2017). Impact of Cytokines and Chemokines on Alzheimer’s Disease Neuropathological Hallmarks. Curr. Alzheimer Res..

[98] Zhang W., Xiao D., Mao Q., Xia H. (2023). Role of neuroinflammation in neurodegeneration development. Signal Transduct. Target. Ther..

[99] di Benedetto G., Burgaletto C., Bellanca C.M., Munafò A., Bernardini R., Cantarella G. (2022). Role of Microglia and Astrocytes in Alzheimer’s Disease: From Neuroinflammation to Ca^2+^ Homeostasis Dysregulation. Cells.

[100] Xie M., Wang T., Feng J., Ma D., Feng L., Hao Y. (2025). Roles of Microglia in Synaptogenesis, Synaptic Pruning, and Synaptic Plasticity in Physiological Conditions and Central Nervous System Disorders. Curr. Neuropharmacol..

[101] Ramos-Triguero A., Navarro-Tapia E., Vieiros M., Martínez L., García-Algar Ó., Andreu-Fernández V. (2025). Machine learning-driven blood biomarker profiling and EGCG intervention in fetal alcohol spectrum disorder. Int. J. Clin. Health Psychol..

[102] García-Domínguez M. (2025). Neuroinflammation: Mechanisms, Dual Roles, and Therapeutic Strategies in Neurological Disorders. Curr. Issues Mol. Biol..

[103] Rosito M., Lauro C., Chece G., Porzia A., Monaco L., Mainiero F., Catalano M., Limatola C., Trettel F. (2014). Trasmembrane chemokines CX3CL1 and CXCL16 drive interplay between neurons, microglia and astrocytes to counteract pMCAO and excitotoxic neuronal death. Front. Cell. Neurosci..

[104] Busayli A.M., Xu W., Raffah G.A., Chen G. (2025). Gut Microbiome, Neuroinflammation, and Fetal Alcohol Spectrum Disorders: Insights from Rodent Models. Biology.

[105] Khan M.A.S., Chang S.L. (2023). Alcohol and the Brain–Gut Axis: The Involvement of Microglia and Enteric Glia in the Process of Neuro-Enteric Inflammation. Cells.

[106] Bhatia S., Drake D.M., Miller L., Wells P.G. (2019). Oxidative stress and DNA damage in the mechanism of fetal alcohol spectrum disorders. Birth Defects Res..

[107] Sowell K.D., Uriu-Adams J.Y., van de Water J., Chambers C.D., Coles C.D., Kable J.A., Yevtushok L., Zymak-Zakutnya N., Wertelecki W., Keen C.L. (2018). Implications of altered maternal cytokine concentrations on infant outcomes in children with prenatal alcohol exposure. Alcohol.

[108] Bodnar T.S., Raineki C., Wertelecki W., Yevtushok L., Plotka L., Zymak-Zakutnya N., Honerkamp-Smith G., Wells A., Rolland M., Woodward T.S. (2018). Altered maternal immune networks are associated with adverse child neurodevelopment: Impact of alcohol consumption during pregnancy. Brain Behav. Immun..

[109] Bodnar T.S., Raineki C., Wertelecki W., Yevtushok L., Plotka L., Granovska I., Zymak-Zakutnya N., Pashtepa A., Wells A., Honerkamp-Smith G. (2020). Immune network dysregulation associated with child neurodevelopmental delay: Modulatory role of prenatal alcohol exposure. J. Neuroinflamm..

[110] May P., Keaster C., Bozeman R., Goodover J., Blankenship J., Kalberg W.O., Buckley D., Brooks M., Hasken J., Gossage J. (2015). Prevalence and characteristics of fetal alcohol syndrome and partial fetal alcohol syndrome in a Rocky Mountain Region City. Drug Alcohol Depend..

[111] Gutherz O.R., Deyssenroth M., Li Q., Hao K., Jacobson J.L., Chen J., Jacobson S.W., Carter R.C. (2022). Potential roles of imprinted genes in the teratogenic effects of alcohol on the placenta, somatic growth, and the developing brain. Exp. Neurol..

[112] del Gobbo G.F., Konwar C., Robinson W.P. (2020). The significance of the placental genome and methylome in fetal and maternal health. Hum. Genet..

[113] Bestry M., Symons M., Larcombe A., Muggli E., Craig J.M., Hutchinson D., Halliday J., Martino D. (2022). Association of prenatal alcohol exposure with offspring DNA methylation in mammals: A systematic review of the evidence. Clin. Epigenet..

[114] Portales-Casamar E., Lussier A.A., Jones M.J., MacIsaac J.L., Edgar R.D., Mah S.M., Barhdadi A., Provost S., Lemieux-Perreault L.P., Cynader M.S. (2016). DNA methylation signature of human fetal alcohol spectrum disorder. Epigenet. Chromatin.

[115] Partida G.C., Laurin C., Ring S.M., Gaunt T.R., McRae A.F., Visscher P.M., Montgomery G.W., Martin N.G., Hemani G., Suderman M. (2018). Genome-wide survey of parent-of-origin effects on DNA methylation identifies candidate imprinted loci in humans. Hum. Mol. Genet..

[116] Passaretti F., Pignata L., Vitiello G., Alesi V., D’Elia G., Cecere F., Acquaviva F., De Brasi D., Novelli A., Riccio A. (2022). Different Mechanisms Cause Hypomethylation of Both *H19* and *KCNQ1OT1* Imprinted Differentially Methylated Regions in Two Cases of Silver–Russell Syndrome Spectrum. Genes.

[117] Eggermann T., Brück J., Knopp C., Fekete G., Kratz C., Tasic V., Kurth I., Elbracht M., Eggermann K., Begemann M. (2020). Need for a precise molecular diagnosis in Beckwith-Wiedemann and Silver-Russell syndrome: What has to be considered and why it is important. J. Mol. Med..

[118] Marjonen H., Kahila H., Kaminen-Ahola N. (2017). rs10732516 polymorphism at the IGF2/H19 locus associates with a genotype-specific trend in placental DNA methylation and head circumference of prenatally alcohol-exposed newborns. Hum. Reprod. Open.

[119] O’Callaghan F.V., O’Callaghan M., Najman J.M., Williams G.M., Bor W. (2003). Maternal alcohol consumption during pregnancy and physical outcomes up to 5 years of age: A longitudinal study. Early Hum. Dev..

[120] Treit S., Zhou D., Chudley A.E., Andrew G., Rasmussen C., Nikkel S.M., Samdup D., Hanlon-Dearman A., Loock C., Beaulieu C. (2016). Relationships between Head Circumference, Brain Volume and Cognition in Children with Prenatal Alcohol Exposure. PLoS ONE.

[121] Troutman J.A., Sullivan M.C., Carr G.J., Fisher J. (2018). Development of growth equations from longitudinal studies of body weight and height in the full term and preterm neonate: From birth to four years postnatal age. Birth Defects Res..

[122] Joya X., Salat-Batlle J., Velezmoro-Jáuregui G., Clavé S., Garcia-Algar O., Vall O. (2015). Prenatal ethanol exposure and placental hCG and IGF2 expression. Placenta.

[123] Ramos-Triguero A., Astals-Vizcaino M., Navarro-Tapia E., Vieiros M., Bastons-Compta A., Martínez L., Andreu-Fernández V., García-Algar Ó. (2026). Cognitive and transcriptomic effects of Epigallocatechin gallate in fetal alcohol spectrum disorders. Sci. Rep..

[124] Kozonis A., Papadoliopoulou M., Margaris I. (2026). Fetal Growth Restriction, Autism Spectrum Disorder and Attention-Deficit/Hyperactivity Disorder-Connecting the Dots: A Narrative Review. Children.

[125] Lussier A.A., Morin A.M., MacIsaac J.L., Salmon J., Weinberg J., Reynolds J.N., Pavlidis P., Chudley A.E., Kobor M.S. (2018). DNA methylation as a predictor of fetal alcohol spectrum disorder. Clin. Epigenet..

[126] Krzyzewska I.M., Lauffer P., Mul A.N., van der Laan L., Yim A.Y.F.L., Cobben J.M., Niklinski J., Chomczyk M.A., Smigiel R., Mannens M.M.A.M. (2023). Expression Quantitative Trait Methylation Analysis Identifies Whole Blood Molecular Footprint in Fetal Alcohol Spectrum Disorder (FASD). Int. J. Mol. Sci..

